# Supplementary material for: Modelling six sustainable development transformations in Australia and their accelerators, impediments, enablers, and interlinkages
Source: Nat Commun. 2024 Jan 18;15:594. doi: 10.1038/s41467-023-44655-4 (PMC10796343; doi:10.1038/s41467-023-44655-4)
Supplement: Supplementary file 1 — Supplementary Information [file 41467_2023_44655_MOESM1_ESM.docx]

Modelling six sustainable development transformations in Australia and their accelerators, impediments, enablers, and interlinkages

Supplementary Information

Contents

[Supplementary Figure 1. BBS Projections for COVID-19 Impacts and government expenditure for BBS and STP projections 2](#_Toc151564706)

[Supplementary Figure 2. Comparison of progress on economic, social and environmental indicators for the two pathways 2](#_Toc151564707)

[Supplementary Figure 3. Detailed results from the sensitivity analysis for the STP trajectory in 2050. 3](#_Toc151564708)

[Supplementary Figure 4. Rising and Declining S-Curves for key technologies and practices – STP trajectory (% adoption) 4](#_Toc151564709)

[Supplementary Figure 5. STP and BBS projections for key indicators relating to T2: Sustainable and Just Economy 5](#_Toc151564710)

[Supplementary Figure 6. STP and BBS projections for key indicators relating to T3: Sustainable Food Systems 6](#_Toc151564711)

[Supplementary Figure 7. STP and BBS projections for key indicators relating to T4: Energy Decarbonization 7](#_Toc151564712)

[Supplementary Figure 8. Overview of the structure of the iSDG simulation model 8](#_Toc151564713)

[Supplementary Figure 9. Baseline model projections and calibration against data: key economic variables 10](#_Toc151564714)

[Supplementary Figure 10. Baseline model projections and calibration against data: key social variables 11](#_Toc151564715)

[Supplementary Figure 11. Baseline model projections and calibration against data: key environmental variables 12](#_Toc151564716)

[Supplementary Table 1. Main exogenous assumptions associated with global drivers for each pathway (BBS and STP) 13](#_Toc151564717)

[Supplementary Table 2. Six transformation storylines 15](#_Toc151564718)

[Supplementary Table 3. Pathway Policy Settings and Assumptions – BBS and STP Policy Levers 29](#_Toc151564719)

[Supplementary Table 4. Evaluation framework of SDG Targets and Indicators (2030) and Transformation Targets (2050) 34](#_Toc151564720)

[Supplementary Table 5. Error analysis of baseline (BBS) simulations for a selection of variables 43](#_Toc151564721)

[Supplementary Table 6. Sensitivity Analysis (SA): sensitivity variables and input ranges 45](#_Toc151564722)

[References 46](#_Toc151564723)

## Supplementary Figure 1. BBS Projections for COVID-19 Impacts and government expenditure for BBS and STP projections

|  |  |
| --- | --- |
| **1(a) Real GDP (AUD bn)** | **1(b) Unemployment rate (%)** |
|  |  |
| **1(c) Total GHG emissions (excluding LULUCF) (Mt CO_2_-eq)** | **1(d) Total general government expenditure for BBS and STP trajectories (current $ bn)** |

## Supplementary Figure 2. Comparison of progress on economic, social and environmental indicators for the two pathways


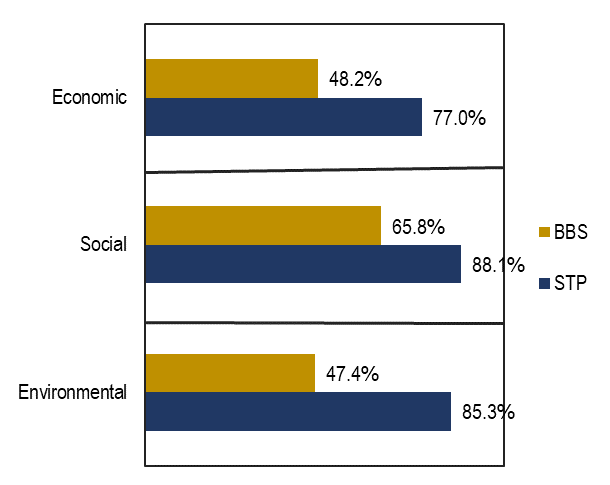


## Supplementary Figure 3. Detailed results from the sensitivity analysis for the STP trajectory in 2050.


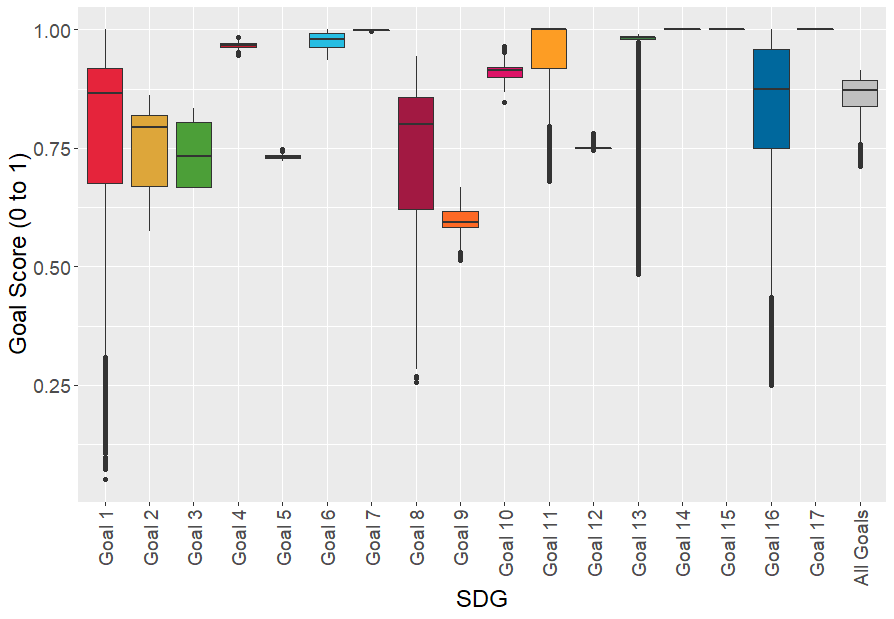


**3(a) Sensitivity analysis results for average progress towards each and all SDGs - projected sensitivity to changes in ten exogenous parameters (6,000 simulations; Latin hypercube). See Supplementary Table 6 for sensitivity parameters and ranges.** Lines in boxes represent the median; the box edges are the lower range (25th percentile (Q1)) and the upper edge (the 75th percentile (Q3)), and the whiskers extend to 1.5 × Q3–Q1). Outliers represent values beyond the whiskers (that is, beyond 1.5 × (Q3–Q1)).


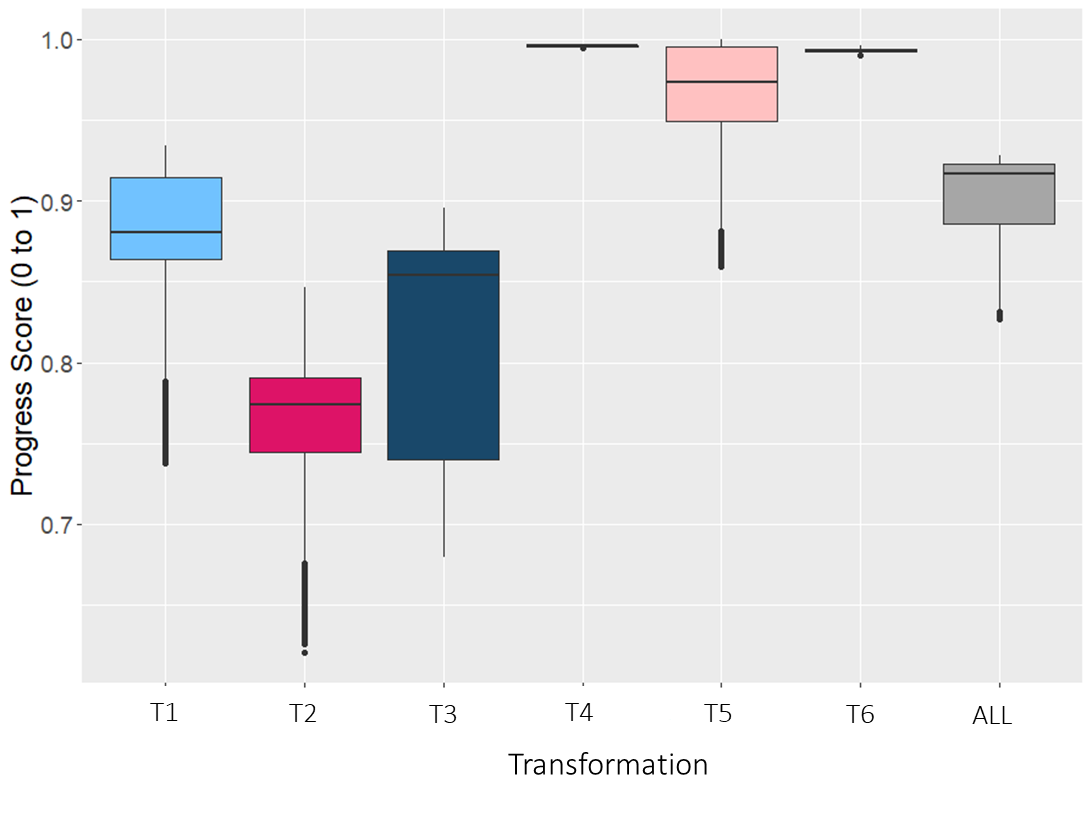


**3(b) Sensitivity analysis results for average progress towards the transformation targets for each transformation and all six transformations – projected sensitivity to changes in ten exogenous parameters (6,000 simulations; Latin hypercube). See Supplementary Table 6 for sensitivity parameters and ranges.** Lines in boxes represent the median; the box edges are the lower range (25th percentile (Q1)) and the upper edge (the 75th percentile (Q3)), and the whiskers extend to 1.5 × Q3–Q1). Outliers represent values beyond the whiskers (that is, beyond 1.5 × (Q3–Q1)).

## Supplementary Figure 4. Rising and Declining S-Curves for key technologies and practices – STP trajectory (% adoption)

*[TFEC = total final energy consumption; BEV = battery electric vehicle; ICE = internal combustion vehicle]*

## Supplementary Figure 5. STP and BBS projections for key indicators relating to T2: Sustainable and Just Economy

|  |  |
| --- | --- |
| **5(a) Projected poverty rate for BBS and STP trajectories to 2050 (%)** | **5(b) Projected Gini coefficient for BBS and STP trajectories to 2050** |
|  |  |
| **5(c) Projected manufacturing production for BBS and STP trajectories to 2050** | **5(d) Projected Material Footprint (MF) per unit output for BBS and STP trajectories to 2050 (kg/USD)** |
|  |  |
| **5(e) Projected Domestic Material Consumption (DMC) for BBS and STP trajectories to 2050 (Mt)** |  |

## Supplementary Figure 6. STP and BBS projections for key indicators relating to T3: Sustainable Food Systems

|  |  |
| --- | --- |
| **6(a) Projected share of harvested area sustainably managed for BBS and STP trajectories to 2050 (%)** | **6(b) Projected per capita fertilizer (N) consumption for BBS and STP trajectories to 2050 (kg/person/year)** |
|  |  |
| **6(c) Projected non-energy agriculture GHG emissions for BBS and STP trajectories to 2050 (Mt CO_2_-eq)** | **6(d) Projected total agriculture production per labour unit for BBS and STP trajectories to 2050 (t/person/year)** |

## Supplementary Figure 7. STP and BBS projections for key indicators relating to T4: Energy Decarbonization

|  |  | |
| --- | --- | --- |
| **7(a). Projected share of renewables in electricity generation for BBS and STP trajectories to 2050 (%)** | | **7(b). Projected share of renewables in total final energy consumption (TFEC) for BBS and STP trajectories to 2050 (%)** |

## Supplementary Figure 8. Overview of the structure of the iSDG simulation model


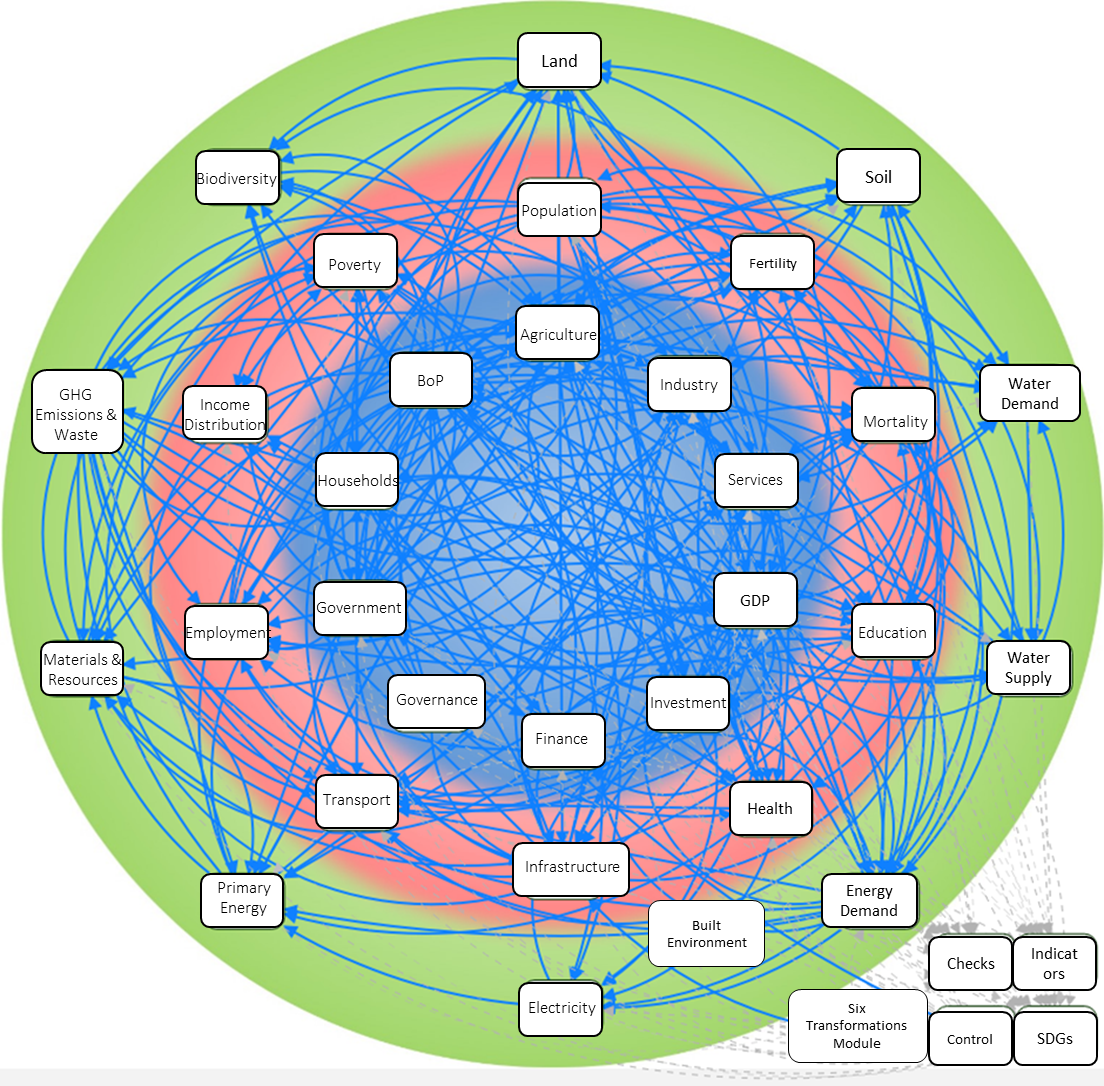


A description of each of the sectoral modules, assumptions and source literature is available in the model documentation ^[1]^. In brief, economic production in the core of the model is divided into agriculture, industry and services (which are further disaggregated by sub-sector) and production is based on Cobb-Douglas production functions which are expanded to include effects of education, health, climate change, governance, infrastructure and other factors on total factor productivity. Investment is endogenously based on public investment, private savings and foreign investment and is allocated across sectors based on the rate of return on investment ^[2]^. Conventional closing rules are used to assure consistency of the macroeconomic framework ^[3]^.

The population module includes endogenous treatment of fertility and mortality along with dynamics for the main factors that influence these variables, while migration is set exogenously. Population is disaggregated into 100 age cohorts and by gender. Births are based on the size of the reproductive age of the population and total fertility rate, while mortality rates are based on income, health, education, exposure to risks and other factors. This also enables estimation of life expectancy.

The government sector is structured according to a standard accounting framework ^[4]^, with tax and non-tax revenues from the households and private sector used for public consumption, investment and transfers. At a functional level, expenditure is allocated for education, health, agriculture, infrastructure, etc. which represent key policy variables that the analyst can set as a desired proportion of GDP or per capita level. Additional financing needs of government are met through domestic and foreign financing.

The land sector simulates land use for different purposes based on standard land cover classifications ^[5]^ and includes endogenous representation of the main factors that shift land between categories (e.g. capital, labour, population growth, protection/restoration); and the soil sector keeps track of macro nutrients and organic carbon density in the soil based on natural nutrients cycles and agriculture production. The water demand sector simulates medium- to long-term trends in water withdrawal by major category (industry, agriculture, domestic), which is met through water supply from renewable sources in the water supply module. The energy consumption module represents major drivers of national final energy demand from economic sectors, residential, transportation and other uses. Electricity demand is met through the electricity generation module which simulates total electricity production from fossil fuels, nuclear and renewables, with capacity expanding to meet expected future demand. The energy supply sector represents primary energy supply of gas, oil, coal, biomass and electricity based on the International Energy Agency’s energy balances ^[6]^. Demographic and economic growth and energy consumption are then used to calculate material consumption, emissions, and waste.

Climate change impacts are introduced through the effects of an increase in global mean temperature (exogenous assumption) on economic productivity, damages to infrastructure and loss of life due to increased risk of natural disasters, and effects of temperature change on biodiversity. The model also allows for exogenous adjustments to precipitation. Expenditure on climate change adaptation can ameliorate negative impacts.

Finally, SDG expenditure, financing and performance modules are used to introduce additional SDG expenditure and financing interventions, as well as calculating SDG performance at the indicator and aggregated target/goal/index levels. A new six transformations module developed for the current study packages policy interventions by transformation and evaluates progress towards the 2050 transformation targets for each individual transformation and all transformations together. The current model includes 52 SDG targets and 80 SDG indicators for 2030 as well as 67 transformation targets for 2050 which are derived from the SDGs and with new target values set for 2050.

##

## Supplementary Figure 9. Baseline model projections and calibration against data: key economic variables


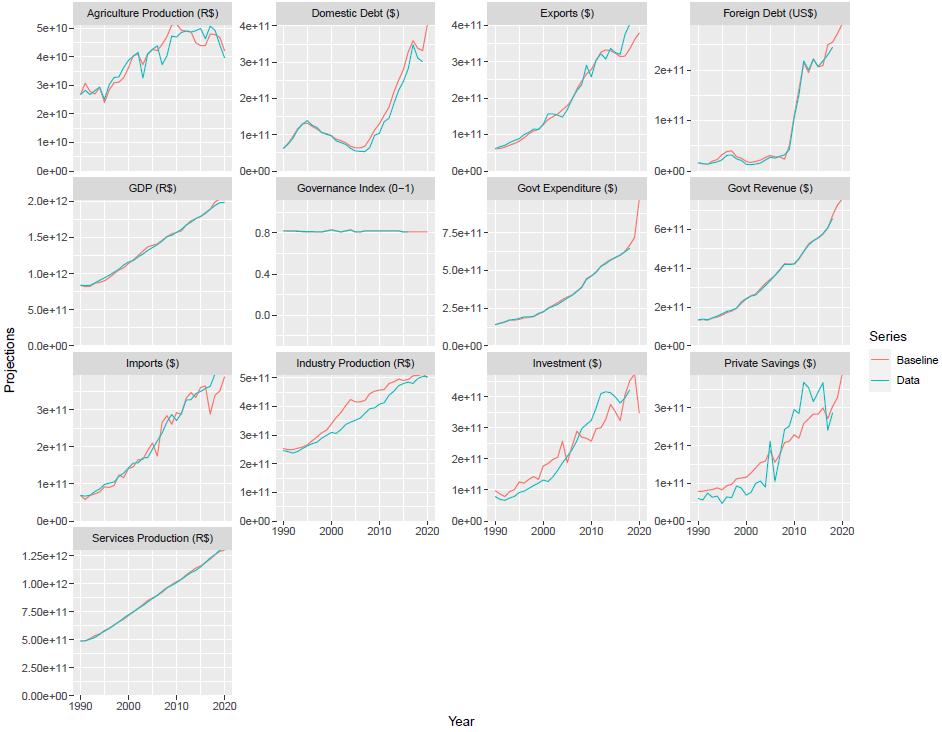


## Supplementary Figure 10. Baseline model projections and calibration against data: key social variables


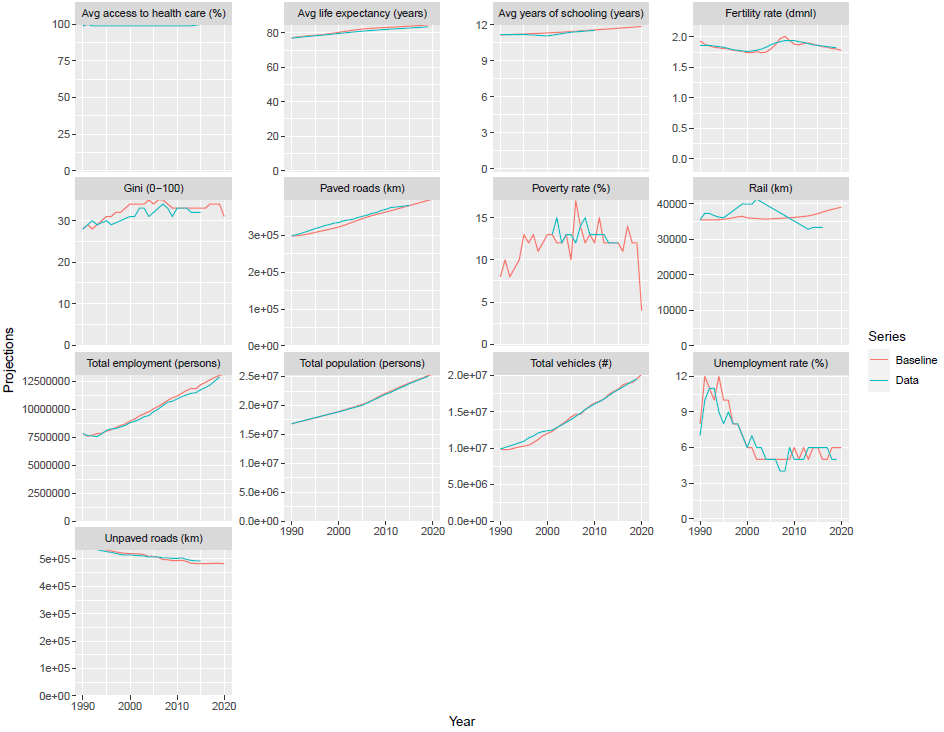


## Supplementary Figure 11. Baseline model projections and calibration against data: key environmental variables


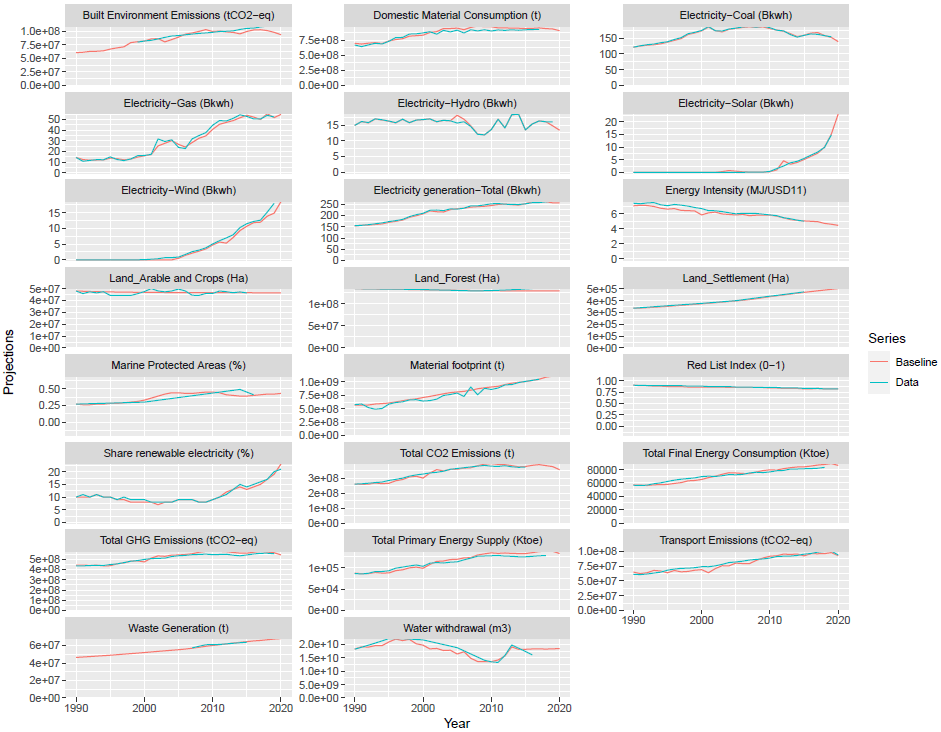


## Supplementary Table 1. Main exogenous assumptions associated with global drivers for each pathway (BBS and STP)

| **Transformations** | **Interventions** | **Build Back the Same (BBS)** | **Six Transformations Pathway (STP)** | **Source/Notes** |
| --- | --- | --- | --- | --- |
| Cross-Cutting Exogenous Assumptions (NB: for individual TP projections, we use the STP assumptions). | Demographics: target net migration per 1,000 population per annum. | 2020: 0  2021: 0  2022: -70%  2023-2030: 7.8 | 2020: 0  2021: 0  2022: -70%  2023-2030: 7.8 | Net migration declines to zero due to border closures and then levels return to pre-COVID average from 2023 onward (~7.8 per 1000 people per annum). Fertility rate unchanged at 1.7. Based on ABS population and migration data ^[7]^. Average value held constant and tested in sensitivity analysis. |
|  | Governance indicators: political stability; government effectiveness; and regulatory quality | Existing trend (0.96, 1.6, 1.9) | Increase in governance metrics (1.8, 1.9, 2) | World Bank Worldwide Governance indicator data ^[8]^ for Australia. STP assumes end of climate wars brings enhanced political stability, regulatory quality and government effectiveness. Effects tested in sensitivity analysis |
|  | Temperature change above pre-industrial | 1.965C by 2050 | Slower increase in average temperature  1.769C by 2050 | SSP temperature projections ^[9]^ and CSIRO projections for Australia ^[10]^. STP assumes temperature reduction based on Paris agreement recommendations with slower growth in global average temperature by 2050. Tested in sensitivity analysis. |
|  | Average yearly precipitation (mm) | No change to 2050  534mm | No change to 2050  534mm | Average yearly precipitation is estimated in the model based on Australian data and projections global modelling projections ^[11-14]^. Precipitation projections show high spatial variability. Average value held constant across trajectories and tested in sensitivity analysis. |
|  | Required increase in adaptation capital per degree increase in temperature (as % GDP) | 0.25 | 0.25 | Adaptation costs estimates based on ^[15-17]^. Costs held constant across trajectories and tested in sensitivity analysis. |
|  | Interest rates on government debt (%) | 3.72% | 3.72% | ABS Government Finance Statistics ^[18]^ and global analyses ^[19, 20]^. Average rate held constant across trajectories and tested in sensitivity analysis. |
|  | Additional decline in trade and commodity prices (fossil fuels) | Decline in mining production in line with demand projections for SSP2 by 2050.  [-3% mining GVA]  [coal -20%; gas and oil -10%] | Accelerated decline in fossil mining production in line with demand projections for SSP1 and national transition away from fossil fuels by 2050. Phase out of coal and oil production by 2050  [-15% total mining GVA]  [coal and oil: -90%; gas: -55%] | Global demand for fossil fuels (coal, gas, oil) is assumed to decline by 2050 using assumptions based on global projections from the SSPs ^[9, 21, 22]^, the IEA’s Net zero by 2050 scenario ^[23]^. and IPCC analysis ^[24]^. Combined with national modelling ^[25, 26]^, and national mining production data ^[27]^ to calibrate assumptions in the model. For SSP2 projections are coal (-20% on 2020 levels), oil and gas (-10% on 2020 levels). For SSP1 (1.9) pathway, global demand projections are coal and oil (-80%) and gas (+15%). The IEA net zero scenario projects a decline in demand for coal (-90%), oil (-75%) and gas (-55%) by 2050. Other global modelled pathways that limit warming to 1.5C project gas consumption to decline with median values of -45% by 2050. For BBS we use SSP2 projections and estimate the impact on mining gross value added (GVA). For STP we use a 1.5C pathway with estimated declines for coal and oil (-90%) and gas (-55%).  Effects on mining GVA are calibrated in the model based on the pace of the green energy transition, with a comparable reduction in total mining GVA in 2050 of -15% compared to reference. Effects of changes in the trade outlook are also tested in the sensitivity analysis. |

## Supplementary Table 2. Six transformation storylines

| **TRANSFORMATION STORYLINE** | **SOURCES** |
| --- | --- |
| **T1. Human wellbeing and capabilities** |  |
| **Progress and Challenges**  In Australia, this transformation which encompasses health, education and resilience could be seen as being at an advanced stage compared to other countries. On the whole, Australians enjoy very good health, are well educated with high levels of tertiary education and a skilled workforce. Average life expectancy is among the highest in the world, supported by a capable, universal healthcare system, however large disparities exist for the Indigenous population. COVID has placed severe strains on hospitals and the health sector, highlighting vulnerabilities and the need for continued public investment to provide ongoing, integrated healthcare for Australia’s rapidly ageing population and for people with long-term conditions. Health expenditure is currently spent primarily on the treatment of illness and disease. Investment in prevention needs to be enhanced in order to achieve a better balance between treatment and prevention in Australia, as outlined in Australia’s Long Term National Health Plan. Australia invests relatively little in early childhood education and remote and disadvantaged communities face significant access and quality gaps. Australia is particularly vulnerable to the impacts of climate change and is on a path towards more frequent, severe and prolonged droughts, heatwaves, floods and bushfires. Costs associated with recent disasters have escalated rapidly, requiring strong action on climate change and strategic investments in adaptation and resilience. Securing sustainable public health financing is challenging as a result of increased government debt and declining revenues which create a structural deficit. Political conflicts remain unresolved around the privatisation or adequate government financing of public services and assets.  **Transformation Impediments**   - **Techno-economic:** new technologies and treatments face higher upfront costs to governments and the public, or require access to devices, digital literacy and supporting infrastructure. Existing investments in infrastructure, technologies and assets reflect a long chain of decisions and balancing of interests across many generations which may amplify vulnerability to disasters and risks but are difficult to change. - **Socio-behavioural**: lifestyles that encourage unhealthy behaviours including fast food, alcohol consumption, sedentary daily routines, or smoking; privacy concerns, beliefs and norms regarding new technologies and data collection may limit uptake of new practices and emerging technologies. - **Political-institutional**: coordination across federal and state jurisdictions and responsibilities; sustainable public health financing in the context of an aging population, high levels of government debt and pressure on governments to undertake budget repair; preventative measures or shifting public investment to lower socio-economic recipients may face strong opposition from existing lobbies including firms selling alcohol, tobacco or gambling or by private education or health operators.   **Positive signs and seeds of change**   - Improved coordination across jurisdictions for the vaccine rollout and preparing hospitals for the pandemic. - Increased access to vocational training through Job Maker Program and additional research funding during COVID. - Recent announcements for new Investments in aged care, early childhood education, childcare and in building resilience to future shocks. - Emerging technological innovations including telehealth, diagnostic and therapeutic tools, wearable technologies, digital platforms, electronic medical records, flexible working arrangements, video conferencing and online learning. - Public awareness improved through active communication from health officials and chief medical officers. - Coalitions built through the development of Australia’s Long-Term National Health Plan, the National Preventative Health Strategy 2021-2030, and Australia’s National Vulnerability Profile.   **T1 Storyline, Enabling Conditions and Interventions to Build Back Better through a Six Transformations Pathway**  The COVID-19 pandemic underscores the importance of having agile and appropriately funded health and education systems which are resilient to shocks including those expected because of climate change. Health and education systems had to adapt quickly during the pandemic and all levels of government responded by setting up structural changes to the system, including expanding telehealth services, GP-led respiratory clinics, and remote teaching and home-schooling platforms and arrangements. Australians rapidly modified their behaviour too, with handwashing, physical distancing, and self-isolating whilst unwell being instilled into the nation’s approach to everyday life. Australia has seen one of the highest global rates of vaccination uptake. The failures of Australia’s aged care system were also brought into focus, resulting in recent announcements of major reforms to the aged care system.  Following the immediate emergency health response, governments and societal actors commit to upgrading Australia’s health and education systems to build back better and ensure that Australia is well-placed to respond to future shocks. Acute public awareness of the health and education system failings and the need for investment in these critical public assets creates the conditions for additional government investment and reforms. A systems-based approach is adopted by public administrators using the SDGs as a roadmap, with funding rebalanced towards encouraging healthy lifestyles, providing the best start in life for children, addressing inequality in health and education systems, and building long-term resilience. Thanks to changes in the way that public expenditure is screened and allocated based on wellbeing (see **T2**), a health and education equity lens is applied to all major government expenditure in these systems.  This results in a shift from a health system fundamentally focused on the treatment of illness and disease, to one that is more focused on prevention and public health as well as addressing weaknesses in aged care. A National Preventative Health Strategy sets clear long-term targets aligned to the SDGs and effectively brings together partners across all levels of government and healthcare providers, professional associations, industry, NGOs, First Nations groups, and individuals. This builds momentum for change, shifts the narrative towards preventative health, and builds public support for new investment and reforms including in early childhood education. Rapid scale-up of digital technologies provide greater accessibility to services, including telehealth, diagnostic and therapeutic tools, wearable technologies, digital platforms, electronic medical records, flexible working arrangements, video conferencing and online learning. Tax reforms (T**2**) provide additional revenue needed for further investments in health and education through until 2030.  Following major bushfire and flooding events, concerns around climate change vulnerabilities are also elevated providing public support for government investment and action to build resilience. Australians see the growing threat for natural hazards to trigger devastating disasters with escalating impacts and expect the government and all sectors to work together to limit the impacts, loss and harm. This generates a shift in narrative around systemic vulnerability and resilience and approaches to address disasters. Building on the momentum gained through the multistakeholder *Profiling Australia’s Vulnerability* initiative led by the Australia government, there is greater cooperation among levels of government, business and societal actors to broker knowledge across domains, unify efforts, and do more than change at the margins.  In line with this narrative, specific quantitative interventions to support this transformation include additional recovery plan expenditure in health, education and climate change adaptation (**Supplementary Table 3**). | ^[28-32]^ |
| **T2. Sustainable and just economy** |  |
| **Progress and Challenges**  Overall, progress on this transformation is still emerging, particularly in achieving a sustainable economy. Until COVID-19, Australia enjoyed the longest period of uninterrupted economic growth of any advanced economy. Productivity growth and increased workforce participation of women have supported these gains. Australia is a leader in a number of industries, such as mining, agriculture, education and health. Despite these strengths, poverty rates remain stubbornly high and increased slightly in recent years (>13%), as social transfers (Newstart) have fallen below the poverty line, leading to growing inequality. Income and wealth inequality remain higher than other OECD countries, with the top 20% of income earners receiving just over 40% of total income. While the bottom 20% receive around 8% total income. Similarly, the top 20% control over 60% of the wealth, while the bottom 20% control less than 1%. Despite temporary relief as a result of government social transfers, COVID-19 is anticipated to have a mainly negative longer-term impact as households have drawn on savings to make it through the crisis, while spiralling cost of living and inflation have placed further pressures, particularly on vulnerable households.  Some of the main elements of Australia’s economic regime have been fiscal conservatism, labour market deregulation, privatisation of state assets, and ‘mutual obligation’ (provision of government funding-support conditional upon recipients being ‘work-ready’). Others include the re-scaling of governance, along with changes to state-based regulation aimed at both stimulating free trade and extending profit-making opportunities for private firms and corporations. However, the sophistication of Australia’s economy has gone backwards, with investment in knowledge based capital and support for collaboration well below innovative nations. There has been limited diversification of Australia’s economy over the past 15 years. The decarbonisation process that will inevitably be required has proceeded slowly not only in sectors with a high material footprint such as primary resources, but across the board. Alongside poor targeting of infrastructure and innovation policies, Australia’s economy has struggled to seize new opportunities to become more resilient and robust for the future. For example, manufacturing has been in decline in Australia since the early 2000s.  Divisions in the Australia’s economic debate are sharp – between economic growth and wider policy goals, between the profits of businesses and the wages of workers, between privatization and public ownership of assets, and between those benefiting from change in inner cities and those struggling to do so in outer suburbs and regions. Australia's economic strategy supports a narrow base of today’s economic winners and hoping that market forces alone will see the benefits ‘trickle down’. This approach will not pull enough people, places and sectors into innovative, higher-value activities. Australia's economic model is struggling to adapt, as public institutions and leaders seem to have lost the capability to build consensus, drive collaboration and improve outcomes. As a result of its COVID-19 response, government has gone heavily into debt, requiring budget repair in coming years. Recent proposals and attempts at tax reform have been derailed due to strong opposition from incumbents and sectors of the community (e.g. mining industry, retirees, investment property owners, media interests etc.).  **Transformation Impediments**   - **Techno-economic**: large sunk investments and inertia in existing dominant industries (e.g energy and resources sectors) crowd out investment in sustainable industries; continued reliance on fossil fuel energy hampers emergence of new industries (e.g. green manufacturing); gaps in skills/capabilities needed for new or emerging sustainable industries; lower return on investment for sustainable alternatives inhibits the scaling up of finance. - **Socio-behavioural:** highly consumptive lifestyles associated with the pursuit of affluence; Australians are aware of inequality, poverty and disadvantage however there is less awareness of prevailing institutional arrangements and decision-making processes that lead to these outcomes, and how to address these. - **Political-institutional**: policy inertia due to powerful industry lobbies (e.g. in energy and resources) with close ties to policy networks which prevent changes to government incentives away from incumbents towards new sectors; the decline of unions in Australia has eroded the capacity for workers to bargain for higher wages; businesses and high-income earners with access to policy makers resist progressive tax reforms intended to favour low income households and adequately finance public services; resistance to tax reform comes from potential ‘losers’ which include businesses, workers, property owners, retirees, shareholders etc. and tax reform becomes a political landmine and is put into the too-hard basket.   **Positive signs and seeds of change**   - Proposals at the state and federal levels to establish wellbeing budgets, building on the New Zealand experience - Recent successes in reconciling contrasting priorities among the business community, governments and union movement including through the Jobs Summit, industrial relations and minimum wages, and legislated and more ambitious climate targets. - A proposed Revenue Summit to bring actors together to discuss tax reform. - Emerging technologies such as green hydrogen and green manufacturing provide green economic opportunities and have support from powerful interests and actors (including Forest Future Industries, Mike Cannon Brookes) - Detailed technical pathways for a decarbonised future economy have emerged through collaborative projects led by stakeholders such as ClimateWorks, CSIRO and Beyond Net Zero - Powerful actors calling for tax reform as well as increasing the Newstart allowance including the Business Council of Australia, Australian Chamber of Commerce and Industry, Union Movement, Australian Council of Social Services - New coalitions in support of increased equality, including the Champions for Change Coalition of leaders in business, government, community and academia for achieving gender equality, advancing more and diverse women in leadership, and building respectful and inclusive workplaces. - Australia’s superannuation sector which manages $3.3 trillion in assets is increasingly aligning investment strategies with social and sustainability objectives, including the SDGs. CBUS recently announced the development of a new Sustainable Development Investments (SDI) framework to align investing with the SDGs, building on leading examples from Europe. - An increasing number of ASX200 companies are undertaking detailed or comprehensive ESG disclosures, with 64% now reporting specifically on the SDGs (up from 10% five years ago). - The Australian Sustainable Finance Initiative brings together a coalition of business interests to develop a roadmap to transform Australia’s financial system to ensure that finance is mobilised and connected to a sustainable future.   **T2 Storyline, Enabling Conditions and Interventions to Build Back Better through a Six Transformations Pathway**  The multiple crises bring the rising cost of living pressures, stubborn poverty rates, and rising inequality into sharp focus. Increased media attention to these issues including by comparison with the more chronic situation in the USA and UK raises public awareness of the poverty and inequality blight and increases broader community support for action. Following the economic fallout from the COVID-19 pandemic, Australia for the first time adopts goals and targets to halve poverty by 2030, reduce inequalities and to close the gender pay gap, amongst others (**Supplementary Table 4**). Building on the successes in New Zealand and emerging proposals in Australia, federal and state governments adopt ‘wellbeing budgets’ which screen major public expenditure to ensure equity and alignment with key social goals. There is a renewed focus on building back better to make sure we emerge from the pandemic with a stronger and more inclusive economy.  Even before COVID-19, the Australian tax system was struggling to meet the needs of the community. COVID creates the ‘burning platform’ needed to ignite the willingness of stakeholders to embrace reform and provides the political momentum for policy makers to challenge and change the status quo. Post-COVID, tax reform becomes a national priority due to high levels of debt accumulated through the response and the need to generate revenue, improve equity, and support growth in green sectors. A national tax reform process led by the Australian government brings together key stakeholders for a National Revenue Summit and builds a coalition of powerful actors in favour of reform, including the Business Council of Australia, Australian Chamber of Commerce and Industry, Union movement, Australian Council of Social Services etc. Building on the successful national Job Summit model which reached compromise between key actors, a National Revenue Summit is convened, resulting in a set of recommended reforms that are promptly implemented by government. Government repeals plans for tax reform which favours high income earners (e.g. Stage 3 tax cuts), with a shift instead towards a more progressive tax system which favours low-income earners. In addition, reforms remove or tighten perverse tax incentives including negative gearing for investment properties and franking credits on self-funded retirees. These reforms are legislated and provide additional revenue from income and profits, international trade and consumption.  Additional revenue funds a broad suite of government investments including an increase in social transfers and redistribution to address poverty and inequality, as well as to support transformations in health and education (**T1**), sustainable food systems (**T3**), energy decarbonization (**T4**), sustainable cities (**T5**) and biodiversity (**T6**). To support the shift towards a sustainable economy, additional investment is made to improve supporting infrastructure needed for green hydrogen and manufacturing industries. Shifting behaviours towards lower consumption and sustainable practices is also supported through changes in education curriculums, information campaigns, and incentives. Stricter regulations and standards on pollution and emissions are also regulated over time to provide incentives and penalties to accelerate technology improvements and behaviour change.  The mission-oriented approach adopted by governments helps to reorient capital to where it can support a thriving Australian society, a healthy environment and a prosperous economy. While governments across Australia – federal, state and local –clearly set a direction for a stronger and more sustainable nation, the Australian Sustainable Finance Roadmap provides the Australian financial system’s contribution to ensuring finance is mobilised and connected to a sustainable future. The SDGs also represent a clear turning point in responsible investing and private capital is increasingly aligned with the SDGs. In particular, Australia’s large superannuation sector throws its support behind the SDGs with all major funds developing sustainable development investment frameworks aligned with the SDGs. This considerably scales up investment in SDG-aligned sectors and divestment from unsustainable industries.  In line with this narrative, specific quantitative interventions (**Supplementary Table 3**) to support this transformation include tax reforms which increase revenue from income and profits, consumption and trade, and increased fiscal pressure on higher income earners with greater distribution to low-income earners. Additional revenue is used to increase subsidies and transfers to households (i.e. increase Newstart) as well as infrastructure expenditure and investments in a green manufacturing transition. Stronger standards and regulations result in increased material consumption efficiency but also reduced industry productivity (**Supplementary Table 3**). | ^[30-40]^ |
| **T3. Sustainable food systems and healthy nutrition** |  |
| **Progress and Challenges**  Australia is internationally recognised as a major food producer and a major quality food supplier. However, access to quality food varies considerably by location and across social and economic groups. Yearly agricultural yields and income can vary significantly, especially those dependent on rainfall, and are vulnerable to natural disasters. Agriculture is largely unsubsidised and export-oriented, with some 60% of total production sold abroad. Australian farmers operate in a very lean financial environment with little government support and limited bargaining power in terms of farm gate prices due to concentration in the Australian supermarket sector. To improve efficiency and increase output, they have adopted the latest technologies and management systems. Neoliberal settings have encouraged specialisation, intensification and economic concentration, as well as the financialization of agriculture where sovereign wealth funds and international investors have purchased large areas of farming land. A strongly concentrated number of multinational corporations hold a large stake in the Australian food system. There is a challenge of succession between farming generations, with young farmers struggling to raise adequate capital to buy farms.  While there are many ways of configuring food systems, the current dominant model organises around an unsustainable growth imperative; the need to produce the highest output at the lowest cost and generate as much profit as possible in the shortest amount of time. This fundamental (yet often hidden) rule is what drives and shapes the industrial food system. Australia’s food system is also organised in a way that makes it energy and emissions-intensive – amounting to 30-40% of Australia’s total GHGs and accounting for the most food-related emissions per capita globally. A large proportion of emissions comes from industrial agriculture, which is heavily dependent on synthetic fertilizers and other fossil fuel derived agrochemicals. The reliance on fertilizers to maintain or improve soil fertility is costly for farmers and disrupts the functioning of soils ecosystems. Soil degradation has also been exacerbated by the widespread removal of deep-rooted native vegetation and replacement with (non-native) annual crops and perennial pastures, which in turn has promulgated habitat destruction, biodiversity loss and water scarcity.  Over the past two decades, irrigation efficiency and water-saving techniques have led to a reduction in agricultural water consumption accompanied by increased productivity. Agriculture uses the largest share of water of any industry.  While Australia produces significantly more food than it consumes, it remains vulnerable to shortfalls in the domestic supply of fruit and vegetables – the very foundation of nutritious diets. The significant concentration of power in the food system also compromises its resilience. Concentration is especially notable at the retail stage, where a small number of companies dominate the market. This imbalance of bargaining power can lead to unfair trading practices, which disadvantages producers, particularly small and medium-size, and generate financial stress among farmers. Over many years, driven by the need to achieve economies of scale (i.e., “get big or get out”), there has been an ongoing restructuring of farming landscapes towards increasing consolidation, concentration and specialisation. These tendencies have come at the expense of many adverse environmental, social and health outcomes. A concentration of power in Australia’s food supply chains is undermining sustainable food systems and the ability of food producers and consumers to enable change. There is little effective regulation to these deficiencies.  **Transformation Impediments**   - **Techno-economic:** sunk investments in agricultural machinery, land and infrastructure and knowledge create vested interests; the current business model involving large-scale land holdings, foreign investment and expected returns, and market concentration in supply chains create barriers to emerging business models based on regenerative, small-scale farming and local supply chains; economies of scale for large-scale farming out-compete small farms; farmers must invest in acquiring skills, equipment, networks, and retail relationships associated with industrial agriculture, and could lose these if they converted to agroecology. - **Socio-behavioural**: modern lifestyles increasingly rely on fast, readily available, convenient ultra-processed foods; the dominant framing or worldview of “sustainable intensification” rather than regenerative or agroecological farming; there is an expectation that food should be cheap and accessible; strong beliefs among scientist, industry, and government elites in the power of science and technology to overcome climate constraints are leading to agroecology being ignored; attention is focused on optimising the current system - **Socio-ecological**: settler colonialism created a particularly agrarian trajectory in Australia which has reshaped landscapes and ecologies and erased indigenous farming systems that could have inspired alternative agriculture. - **Political-institutional**: existing government policy settings, research and extension services, and retailer bulk supply preferences favour large-scale industrial farming and incumbent firms; there is a lack of support and uneven playing field for local farmers; corporate concentration of power and vested interests prevent change and hamper innovation; supermarket duopoly has the power to determine the conditions of supply chains; a web of interlocking market and political incentives tailored to large-scale farming reinforces the use of industrial methods.   **Positive Signs and Seeds of Change**   - Growing number of farmers willing to transition to agroecological practices through proactive investment and capacity building. - High-Level Panel of Experts on Food Security and other independent bodies concerned with the sustainability of our food system are calling for a radical transformation by boldly reshaping the underlying principles that organise it from production to consumption. - Various emerging initiatives in Australia are promoting food systems transformation, including Resilient Foodbowl, Ripe for Change, CSIRO Future Food Systems and CRC, Regenerative Food Systems Investment Australia, and RegenWA. - Public awareness of the need for food systems transformation is increasing as a result of popular books (e.g. Call of the Reed Warbler) and films (e.g. Regeneration). There is also increasing public awareness of healthy diets and lifestyles and growing demand for organic and fresh foods. - Governments and their partners are also moving ahead, including VicHealth’s recent Consensus Statement – Towards a Healthy, Regenerative and Equitable Food System in Victoria, which provides a call to action for state and local governments on the urgent need for food systems transition. - Many of the players in the regenerative food systems movement are trying to establish new enterprises and business models which meet and build upon the growing demand for more sustainable food, including farm to plate local produce delivery, and considerable growth in farmers markets. - Many emerging technologies have the potential to disrupt the food system and create new products and value, including plant-based meat alternatives, circularity in food systems, vertical agriculture, livestock/seafood substitutes, seaweed for food/feed, microbial proteins, big data and IoT, enteric fermentation. - Globally, the Food and Land Use Coalition is working to develop integrated pathways for sustainable food and land use, aligned with the SDGs, and have identified 10 critical transitions required to bring climate change under control, safeguard biological diversity and improve diets and food security. This work is being used to inform the development of Australian pathways for sustainable food and land use through the next phase of the Land Use Futures program, led by ClimateWorks Australia (working within the Monash Sustainable Development Institute), Deakin University and CSIRO.   **T3 Storyline, Enabling Conditions and Interventions to Build Back Better through a Six Transformations Pathway**  The bushfire devastation, unprecedented floods and COVID-19 shine a bright light on the extreme shortcomings in our food system. Not least, the extensive disruption and economic impact of COVID-19, including multiple lockdowns, business closures and cutbacks of social welfare (after JobSeeker supplements ended) meant that food insecurity became a more prevalent and growing issue. The 2021 State of Environment Report confirms again that the state and trend of Australia’s environment are poor and deteriorating as a result of increasing pressures including from agriculture and land use. Instead of jumping into ‘solutions mode’, governments and stakeholders take time to ask questions and reach shared agreement on the desired characteristics of Australia’s future food system, guided by the SDGs. A collaborative problem-solving approach is taken to identify enabling conditions that would unlock a transition towards that healthy, regenerative and equitable future. Change comes through a process of ‘massification’ – a process that leads ever-greater numbers of farmers to practice agroecology over ever-larger territories and which engages more people in the processing, distribution, and consumption of agroecologically produced food. Key drivers of change include the crises, coalescing social organization, effective agroecological practices and solutions, external allies, and favourable policies.  A regenerative agriculture movement gains momentum with impetus from popular books and films (‘*Regenerating Australia’*). Emerging business models such as farm to table distributors, the proliferation of local farmers markets and changing preferences for healthy diets and organic produce support momentum for change. Many emerging technologies begin to disrupt the food system including plant-based and laboratory proteins, feed substitutes, big data and IoT. These solutions provide viable alternatives that are pushed by governments, business and civil society stakeholders who are calling for an urgent food systems transition.  Building on emerging initiatives supporting regenerative and agroecological farming across all states, a sustainable food systems movement largely driven by non-government players such as grassroots communities and not-for-profits, universities, philanthropy and some parts of industry builds momentum for decisive action and investment. Shifting narratives and values around healthy diets and lifestyles begins to erode support for current incumbent firms, with people seeking out local farmers markets and delivery alternatives. The government takes a leadership role building on its commitment to a sustainable and just economy, devising an integrated and well-resourced pathway forward. After initially developing a plan to safeguard acute food security, a national Working Group shifts attention to prioritising action towards ensuring long-term food security –articulating a shared vision and principles and actions that governments, communities and individuals can take to mobilise a transition towards a healthy, regenerative and equitable food system in Australia. This reorients the food system around new goals and targets and an agroecological model which orientates around producing diverse, nutritious foods, regenerating ecosystem processes (rather than running them down) and enhancing farmer livelihoods. This is not a new model – it is built upon a foundation of traditional, place-based and indigenous knowledge. As a result of this systems approach, multiple benefits are simultaneously realised. These include more diverse and resilient agroecosystems that conserve water, build soil health, restore habitats, promote biodiversity and reinvigorate food economies across rural, peri-urban, and urban communities through job creation.  Framed by the SDGs, government, business, farmers, academia and communities collaborate to build a more sustainable and inclusive food system, rapidly accelerating the widespread uptake of agroecological and regenerative farming practices and drawing in as many farmers as possible. Governments lead the way by offering incentives and innovative investment options to pay farmers and land managers for regeneration and to support education, extension services, and peer-to-peer learning networks. Adverse incentives and regulatory barriers to regenerative farming are also identified and removed. Regenerative farming approaches are also accelerated by scaling up existing initiatives that connect food providers with consumers through transparent supply chain models, made up of many and diverse food enterprises such as food hubs, coops, community-supported agriculture and farmers markets. Rebuilding a robust social welfare system (T**2**) also helps farmers to maintain their livelihoods while transitioning to agroecological practices.  Sustainable agricultural practices begin to reduce GHG emissions from agriculture which are predominantly from livestock. The growth in consumer preferences for plant-based and lab-based proteins places downward pressure on domestic and global demand for meat products. Vaccinations, feed supplements and other improvements are made to drastically reduce emissions from enteric fermentation over the longer term, reducing methane emissions. Similarly, improved systems for the management and storage of fertilizers, composting and pelletising manure, and precision agriculture reduce GHG emissions associated with cropping. These reductions are in line with ClimateWorks deep decarbonisation 1.5C pathway. Intensive silvopasture with a combination of trees and pasture further sequesters CO_2_ emissions.  In line with this narrative, specific quantitative interventions (**Supplementary Table 3**) to support this transformation include additional government investment in sustainable agriculture training, extension services and water management, and reduction in emissions associated with livestock and crops. | ^[41-53]^ |
| **T4. Energy decarbonization with universal access** |  |
| **Progress and Challenges**  While access to electricity is near universal in Australia, the retail price of electricity has more than doubled in the past decade. This means Australians now pay higher electricity prices than most other OECD countries. Energy affordability has become a major problem facing many Australians, particularly those on lower incomes. Australian energy exports have increased over 20-fold in the past four decades, and now amount to about three times Australia’s domestic primary energy consumption. Australia’s energy exports are dominated by coal, gas and uranium. While Australia currently exports no renewable energy, opportunities exist to develop this activity in the future.  Australia remains highly dependent on fossil fuels and the share of renewables in final energy consumption increased marginally since 2000. The share of renewables in electricity remained stable up until 2010. Since then, it has more than doubled as a result of the Australian Government’s Renewable Energy Target and state–based actions which are now driving substantial investment in renewable electricity generation which accounts for 23% of electricity generated by 2020. In parallel to investments in large scale renewable electricity assets, there have been strong increases in small scale systems, driven by solar PV installation. The number of small-scale solar PV installations are now equivalent to nearly 20% of households in Australia, making it one of the highest penetrations in the world. This has been driven by government subsidies and feed-in tariffs and rebates which have incentivized household investment. Rapidly falling costs in renewable generation technologies in recent years have also been a key driver of more rapid uptake, as have public concerns about the impact of climate change and an aging coal generation fleet. Government projections suggest Australia will generate 48% of electricity from renewables by 2030.  Under the Paris Climate Agreement, Australia originally committed to reduce total greenhouse gas emissions to 26–28% below 2005 levels by 2030 (from 597 Mt CO2 to 441 Mt CO2). This was subsequently raised to 40% in 2022 with a change in government. Australia still has the highest per capita emissions of any OECD country. Although the makeup of emissions is different—with large increases in emissions from industry and transport, and lower emissions from land clearing—Australia’s emissions are similar now to what they were in 2000.  A key impediment to the acceleration of the energy transition has been policy uncertainty. The failure to extend the Renewable Energy Target beyond 2020 and the introduction and then repeal of a legislated carbon price have contributed to this uncertainty. Australia also currently provides higher subsidies for fossil fuel use than other OECD countries, currently running at over three times the OECD average. These subsidies create artificial discounts across a range of products and tend to distract focus from the importance of investment in rail infrastructure and alternative sources of energy.  Australia has virtually unlimited supplies of sun and wind for renewable energy generation and is particularly vulnerable to climate impacts, however is still heavily reliant on coal and natural gas for electricity generation which gives enormous potential power to the incumbent energy sector. Australia has abundant quantities of coal and natural gas and is the world’s largest coal exporter and has built enormous LNG export infrastructure over the past decade. This creates considerable inertia in the energy system. Australian energy incumbents have an almost thirty-year history of success in blocking, weakening, delaying or shaping policy responses to climate change. They have ensured that policies contained significant caveats and loopholes to allow ‘business as usual’. Climate change and the so-called ‘climate wars’ have been intimately involved in the downfall of at least three governments. Incumbents have worked to ensure that National Electricity Market (NEM) rules favour large, centralised fossil fuel generators, making market entry harder for decentralised and renewable sources. To achieve this, they have used lobbying, supplemented with economic modelling, ‘hearts and minds’ publicity campaigns which either burnish their industries or attack proposals for change, and the creation of organisations that will make their case to policy networks or beyond. In addition, they have striven to slow the growth of alternative sources of electricity generation, while supporting the expansion of fossil fuel infrastructure and shaping the NEM to suit the needs of centralised fossil-fuel generators. Incumbents also stand accused of having deliberately and consistently over-estimated future electricity demand to build state-funded infrastructure, so-called ‘gold-plating’ of the electricity grid.  **Transformation Impediments**   - **Techno-economic**: large sunk investments in centralised electricity generation and the energy resources sector create powerful vested interests; low-cost, high-performance characteristics and economies of scale associated with fossil-fuel energy create barriers to investment in renewable alternatives; existing grid infrastructure has been designed for centralised rather than decentralised energy, with large investments in transmission and storage needed; those who own the infrastructure – for extraction, distribution and retail – are keen to continue profitable business and have acted extremely effectively in their defence. - **Socio-behavioural**: lifestyles have become structured around high-consumption of readily available, high-performance and reliable energy; a concerted campaign of misinformation and issue minimisation which has created confusion around causes and solutions and diffused public support and momentum for change. - **Political-institutional**: existing subsidies and policies continue to support fossil fuel consumption while also encouraging renewables; NEM rules favour large, centralised fossil fuel generators; powerful vested interests in the energy and resources sector have sought and succeeded in preventing policy shifts and stifling innovation; firms, employees, and regional communities in sectors such as coal mining have resisted the transition due to potential job losses; powerful lobbies have successfully captured and infiltrated government to prosecute their commercial interests; a key strategy by business incumbents has been concerted and coordinated lobbying of selected ministers and senior bureaucrats, almost always backed up by economic modelling.   **Positive Signs and Seeds of Change**   - Despite almost 30 years of resistance, the energy transition in Australia has begun to accelerate with record large-scale wind and solar energy installations, new storage, and network infrastructure gaining speed. The technologies needed for the transition are readily available and increasingly economically competitive. - Progress in the last five years has closed the technical gap with key technologies crossing tipping points which mean that achieving zero emissions is possible in many sectors. Mature technologies capable of achieving zero emissions already exist in many sectors and technical obstacles have been overcome. Investment in renewables is rapidly scaling, with emerging projects such as Sun Cable and green hydrogen providing promising largescale investment opportunities. - Community concerns about the impact of fossil fuel generation on carbon emissions, along with technology changes and an ageing coal fired generation fleet, are among factors that have driven and continue to drive Australia’s energy market transition. - National emissions reduction targets have recently been upgraded to 40% reduction by 2030, and with a firm commitment to net zero by 2050. Detailed technical studies and roadmaps developed by ClimateWorks, CSIRO, Beyond Zero Emissions and their partners provide pathways to decarbonise Australia’s energy sector. - In electricity, zero-emissions technologies are readily available although not yet deployed at sufficient scale. In particular, large- and small-scale renewable electricity generation (supported by new storage capabilities and demand management measures) can fully decarbonise Australia’s power supply. The increased uptake of new technologies worldwide has led to significant cost reductions, with new large-scale renewable electricity generation now less expensive than new fossil fuel generation, and battery costs per kilowatt hour 80% cheaper than in 2010. - Globally and in Australia, major corporations, investors and governments are already moving to align their strategies with the goal of net zero emissions. - All Australian states and territories are now aiming to achieve net zero by or before 2050. In addition, Australian capital cities and local governments are increasingly setting net zero emissions targets for their communities. State government interventions in energy systems are accelerating the transition (e.g. the South Australian government’s investment in large-scale battery storage). - Rising public and political support for a re-nationalised electricity grid and supply and open discussions of similar initiatives in public transport   **T4 Storyline, Enabling Conditions and Interventions to Build Back Better through a Six Transformations Pathway**  Following the Black Summer fires and unprecedented floods, public support for action on climate change reaches new levels and powerful actors call for a green recovery from COVID-19. Bottom-up political movements and collective action see a shift in politics away from the status quo in support of decisive policy on climate change, disrupting incumbents and providing a window of opportunity to end the climate wars. A powerful coalition of political, business, community and union actors agrees on shared ambitious mitigation targets for Australia, supported by a clear plan for policy and investments needed over the next 10 years to accelerate the transition towards 100% renewables. Longer-term plans are developed to reduce demand and tackle emissions in hard-to-abate sectors. By setting ambitious targets immediately, decision makers focus attention on new solutions and prevent further missed opportunities in technological investment. Stakeholder activism and divestment and hostile-takeover of fossil fuel assets by powerful commercial actors in Australia (e.g. Mike Cannon-Brookes and Brookfield) result in an accelerated phase out of fossil fuel generation. Coalitions continue to build momentum, including between government and leading business lobbies such as the Business Council of Australia, bringing together allies in the broader business sector where an increasing number of businesses have committed to achieve net zero emissions by 2050, including software company Atlassian, property companies Dexus and Mirvac, resources company Rio Tinto and Qantas airlines.  Existing roadmaps for deep decarbonisation developed by ClimateWorks and CSIRO with support from a wide range of government and commercial partners shape government policy and private investment across the energy, built environment (**T5**), transport (**T5**), green manufacturing **(T2**) and agriculture and land sectors (**T3 and T6**). This results in accelerated deployment of mature and demonstrated zero-emissions technologies, and the rapid development and commercialisation of emerging zero-emissions technologies in harder to abate sectors. The strategy is built around four key pillars: energy demand and waste reduction; 100% renewable electricity; electrification and fuel switching from fossil fuels to zero or low emission alternatives; non-energy emissions reductions and offsetting of residual emissions. With governments, business and individuals mobilising to achieve faster change, progress towards net zero is turbocharged. Governments drive emissions reductions through legislation, regulation and incentives; businesses and individuals reduce emissions through their consumption, investment and advocacy; and technologies and innovation continue to provide the solutions and new ways of working. From 2030 to 2050, the implementation challenge shifts to zero-emissions solutions for long-haul transport, agriculture, and industry, which need to be the focus of accelerated RD&D investment over the period to 2030.  In line with this narrative, specific quantitative interventions (**Supplementary Table 3**) to support this transformation include additional investment in small- and large-scale renewable energy including solar, wind and hydro and supporting infrastructure, additional investment in industry energy efficiency, fuel switching from fossil fuels in industry, more rapid phase out of coal and gas electricity generation, and reductions in emissions from waste and industrial processes. | ^[30, 31, 37, 48, 54, 55]^ |
| **T5. Urban and peri-urban development** |  |
| **Progress and Challenges**  Australian cities are well-serviced in terms of access to drinking water, sanitation, waste and transport however average household bills are increasing steadily. Prolonged droughts and water stress have resulted in investments in water efficiency and demand reduction, with per capita water extraction declining substantially since 2001. Areas of green space in cities have been increasing, with large green spaces in major capital cities, and air pollution levels are comparatively low.  However, the pressures of population growth on Australia’s ever-sprawling cities are increasingly evident. Australian cities are responsible for a large proportion of the country’s demand for resources. The growing urban footprint in Australian cities is often accompanied by high rates of car dependence; this results in flow-on effects such as increased emissions and air pollution, adverse health effects, and frustration over lost time. Congestion keeps rising while access to jobs and services (for many) is diminishing, resulting in environmental degradation and growing social exclusion and inequality. Governments are finding it difficult to fund the infrastructure and services required to keep up with population growth, while development, production and consumption patterns are socially and ecologically unsustainable. Housing affordability is a serious concern in Australia, with the share of income spent on rent or mortgage repayments steadily increasing and COVID expected to have a negative impact. The passenger car remains the dominant mode of transport in Australia, accounting for 64% of passenger travel. The share of electric vehicles remains low.  In Australia, urban development and practices are not guided by a clear long-term agenda – cities pursue their goals in isolation without a clear national vision that connects their efforts, shares knowledge, builds capacity and creates innovation. Lack of leadership, agreed goals and targets leads to short-termism in decision making. Transformation in cities is countered by processes of inertia or path dependence (the self-reinforcing processes that guide the development of a system) and lock-in (the historically evolved system state and its physical embodiment that cannot be changed easily). For example, the co-evolution of large interdependent technological networks, social institutions, cultural practices and incumbent businesses that support and benefit from system growth.  **Transformation Impediments**   - **Techno-economic**: existing physical infrastructure in cities creates inertia when it comes to urban change; high up-front costs associated with retrofitting existing infrastructure and assets and purchasing higher-cost sustainable alternatives. - **Socio-behavioural**: Citizens’ consumption behaviours diverge from desired values (e.g. on sharing and waste) and growing urban social issues and disadvantage are often hidden from view (e.g. inequality, poverty, unemployment); social preferences to live in large houses and for personal car ownership promote urban sprawl and are reinforced by existing urban design. - **Political-institutional**: Lack of consistent national government direction and conflicting policies and governance across levels and sectors; conventional planning focused on form and function rather than place making; there is no single authority responsible for planning or infrastructure in Australia’s cities, and local authorities have little agency to significantly change these aspects; government policies and investments preference road infrastructure and promote urban sprawl; developers and other vested interests (e.g. benefitting from peri-urban green field developments) may resist change.   **Positive Signs and Seeds of Change**   - Participation of Australian cities in various organisations and initiatives such as the Global Parliament of Mayors, C40, ICLEI and Compact of Mayors, who are all becoming increasingly prominent in global climate change governance. The recently established Cities Power Partnership program of the Climate Council accelerates emission reductions in Australian cities and councils. - Urban planners and governments are recognising the need for change and a more collaborative, whole-of-system approach, with place-based outcomes that can build greater resilience and regenerate our urban areas. - Research initiatives such as FutureEarth and the CRCs for Water Sensitive Design and Low Carbon Living are providing solutions and pathways to sustainable cities. For example, Decarbonising the Built Environment: Charting the Transition is a key outcome for the CRC for Low Carbon Living involving partners from government, business, civil society, academia, and international organisations. This includes the Visions and Pathways 2040 project which developed visions, scenarios and pathways for transforming Australian cities to achieve rapid decarbonisation as well as increased resilience in the face of climate change. - There have been some institutional advances for improving coordination across cities and regions in land use, infrastructure, and transport planning on the part of state governments. The Greater Sydney Commission (GSC), for example. - Cities contribute to fostering new innovations and industries, for example large, comparatively wealthy, urban populations provide a context that can support new market development, aiding commercialisation and adoption of technologies and practices. - A greater proportion of people living in Australia’s major capital cities report concern about climate change. Growing political support for alternative parties and independent candidates is indicative of changing norms and values and voting trends. - Many councils who are keen to take action on sustainability are frustrated by the lack of clarity or consistency in the state and national policy, and local councils who are willing to push ahead of their states on targets and actions have formed their own alliances. - Most of the technology solutions required to achieve zero emissions in the built environment (for instance, deep energy efficiency and the electrification – with renewable energy – of power, heating and water services) are mature and commercially competitive or have been demonstrated at scale. Energy-efficient technologies continue to become cheaper and more effective (however, consumer uptake has not kept pace with technology improvements). - In transport, the extraordinary fall in battery costs (together with supportive government policies) means some 3 million electric vehicles are already being driven throughout the world, and may be reaching a global tipping point. The electrification of passenger and freight transport (together with the optimisation of travel needs, mode-shift, and the transition to renewable electricity) demonstrates how the transport sector can be cost-effectively decarbonised. - National Cities Performance Framework provides access to data and benchmarking to compare the performance of cities across a range of metrics, many of which are aligned to the SDGs.   **T5 Storyline, Enabling Conditions and Interventions to Build Back Better through a Six Transformations Pathway**  Australian politics to 2021 was partly a contestation between the different tiers of government and between generations. Paralysed, dysfunctional federal politics saw attention shift to the states and cities – particularly for leadership on renewable energy, energy efficiency, and green industries and jobs. Australia’s climate-leading municipalities had become frustrated by the jurisdictional limits on their abilities to meet the bold, progressive emissions reductions targets they had set in the early 2000s. Even with the strong leadership and major investments they were now making, there was only so much they could do alone. As the homes of many economic and cultural leaders and powerful actors, they set about driving changes to corporate behaviour and turning up the heat on state and federal governments. As the ‘climate-action’ population centres of Australia, the major cities quietly took steps to enable and strengthen the bottom-up movements calling on ambitious goals including a just transition towards net zero.  Building on initiatives such as the CRCs for Low Carbon Living and Water Sensitive Cities, the Australian Housing and Urban Research Institute (AHURI), CSIRO Urban Living Labs, and the Australian Urban Research Infrastructure Network (AURIN), a national framework of local visions and plans are developed and tailored for each city including goals and targets. These are co-designed with multiple stakeholders representing all governance levels of urban decision-making, and inform sustainable development pathways that bring visioning processes to life. This improves community and sectoral buy-in, guiding policy measures, generating investments, and raising awareness. At a national level, The National Cities Performance Framework is aligned to these strategies and supports benchmarking and experience sharing.  Targets and plans support rapid decarbonisation over the next few decades, provide long-term strategies and address integration of policies across sectors. Common focus areas of action are energy use (buildings), energy production (**T4**), transport, urban development, waste/resource utilisation and city administration. Focus is initially on energy use in buildings and transport which constitute the largest sources of GHG emissions, followed by emissions from waste treatment. National, state and city governments support investments in electrification of buildings, electric vehicle subsidies, tax rebates and charging infrastructure. Working with community partners, a widespread campaign to shift behaviours towards sustainable practices is implemented, including education, marketing, awareness raising, and incentives to reduce, reuse and recycle waste, consume local produce, and support local food systems (**T3**), utilise public transport, and encourage healthy lifestyles built on sustainable diets, green space, thriving cultures and inclusive decision making. The implementation of tax reforms (including removal of negative gearing) (**T2**) and large investments in social housing improve the affordability of housing.  In line with this narrative, specific quantitative interventions (**Supplementary Table 3**) to support this transformation include investments in vehicle and energy efficiency and demand reduction, electrification of residential and commercial buildings, increase in the share of timber building structures and timber resource recovery, reductions in waste generation and non-recycled municipal waste, subsidies and tax rebates for battery electric vehicles/bikes/trucks/buses, investment in charging infrastructure, the forced phase out of internal combustion vehicles, and additional investment in adaptation and resilience. | ^[30, 31, 48, 56-60]^ |
| **T6. Global environmental commons** |  |
| **Progress and Challenges**  While Australia is currently meeting Aichi Biodiversity Target 11 in achieving protection for 17% of terrestrial areas and inland waters, many of our bioregions are underrepresented with the majority experiencing downgrading, downsizing or de-gazettement. More than 20% of Key Biodiversity Areas (KBAs) are not represented in protected areas and only a small proportion of KBAs are fully represented—with the rate of improvement of complete coverage slowing down. The status of Australia’s species—as adjudicated by assessment of vertebrates and cycads in the Red List Index and a wealth of other scientific studies—is one of decline. Mammals are in a particularly perilous state, and the outlook for threatened species is generally not promising. Australia’s marine protected areas exceed the 10% Aichi Target, however the share of no-take protection zones remains relatively low. Assessments of reef systems along the eastern and western coasts of Australia show declines in percentage reef cover and increases in the extent and severity of bleaching over the past several decades. Overall, Australia is not only failing to protect and restore its ecosystems, but is moving in the wrong direction, and with little evidence of impending action to reverse the trend.  Overall, the state and trend of the environment of Australia are poor and deteriorating as a result of increasing pressures from climate change, habitat loss, invasive species, pollution and resource extraction. Changing environmental conditions mean that many species and ecosystems are increasingly threatened. Multiple pressures create cumulative impacts that amplify threats to the environment, and abrupt changes in ecological systems have been recorded in the past 5 years. The Australian Government’s 2015 Threatened Species Strategy 5-year action plan achieved partial success by improving the trajectories of 24 priority species by 2020, but many did not show improvements and, overall, the number of listed species has grown by 8% since 2016. The number of listed entities will increase substantially in coming years as a result of the 2019–20 bushfires. The inability to adequately manage pressures continues to result in species extinctions and deteriorating ecosystem condition, which are reducing the environmental capital on which current and future economies depend. Social, environmental and economic impacts are already apparent.  The management of Australia’s environment involves many components and many organisations. Most land management is undertaken by landholders, Indigenous communities, government, non-government organisations, industry and volunteers, but only a very small proportion of this management is undertaken with the direct purpose of maintaining or improving environmental values. Australia currently lacks a framework that delivers holistic environmental management to integrate disconnected legislative and institutional national, state and territory systems, and break down existing barriers to stimulate new models and partnerships for innovative environmental management and financing. Immediate action to reduce carbon emissions would result in reduced pressures and improved trajectories for most aspects of the environment. There is a growing recognition of the need to engage Indigenous peoples and knowledge in caring for country, however there is a complex web of government laws and agreements that relate to Indigenous people and the environment, and overall, they are not adequate to deliver the rights that Indigenous people seek.  **Transformation Impediments**  The barriers associated with this transformation are largely linked to other key transformations – particularly T2, T3, T4 and T5 – see above.   - **Techno-economic**: dominant patterns of economic activity and consumption and production generally fail to address the costs to nature, resulting in urban sprawl, land clearing, habitat loss, pollution. - **Socio-behavioural**: modern lifestyles create a separation from nature and how we feel about nature is often anchored in childhood experience; highly consumptive lifestyles are a key driver of pressures on the natural environment. - **Socio-ecological**: settler colonialism has reshaped landscapes and ecologies massively and led to the loss of considerable indigenous knowledge on ecological management. - **Political-institutional**: policies and investments largely fail to recognise the value of ecosystem services and fail to cost environmental externalities; vested interests who benefit from these ‘free services’ resist costing them into their business models; disconnected legislative and institutional systems across different levels.   **Positive Signs and Seeds of Change**   - The recent national State of the Environment Report 2021 explores the links between human wellbeing and the environment and is increasingly aligning its analysis with the SDGs, including outcome-based assessments which provide a common framework that can be used by industry and states and territories. - Australian individuals, communities, nongovernment organisations and businesses are engaging with nature and supporting biodiversity and heritage. Successful on-ground actions include the work of Indigenous rangers, citizen science, and restoration actions at many scales, providing opportunities that deliver benefits for people and Country. Indigenous people are seeking greater participation in Australia’s environmental management system. - Substantial data about the environment are becoming more available. Over the past 6 years, the National Environmental Science Program and the National Collaborative Research Infrastructure Strategy have become important sources of information for state of the environment reporting, and have provided critical funding for research informing policy and on-ground management of the environment. - Land Use Futures are exploring how Australia can live within limits set by the global planetary boundaries and understand Australia’s contribution to global health. - Globally, momentum for nature-positive economies is growing, with organisations including the World Economic Forum and the United Nations promoting this as a global goal for nature. - Both regulation and accelerating market demand is also increasing pressure on businesses to act. Corporate and institutional investors are demanding nature-related disclosure and the setting of science-based targets for nature. Regulators are also taking more ambitious steps, building natural capital accounting into land management and market valuation. - Initiatives such as the National Capital Roadmap are advancing tools and partnerships to put in place systems to make more informed decisions about the environment, with many emerging approaches to measuring and valuing natural capital. - Nature-based solutions such as carbon forestry provide the opportunity to improve landscapes and address climate change.   **T6 Storyline, Enabling Conditions and Interventions to Build Back Better through a Sustainable Wellbeing Transformation**  The unfolding biodiversity crisis is exacerbated by recent catastrophic bushfires and floods. Through the COVID-19 lockdowns, an increasing appreciation for nature emerges in the population as people seek the great outdoors for relaxation and recreation and thousands relocate from major cities to regional areas in search of a tree change. Building on the experience in the latest State of Environment report where indigenous and non-indigenous people worked side by side to combine knowledge, a more holistic understanding of Australia’s environment is enabled which feeds through to new partnerships to more effectively manage Australia’s natural assets. Connections between people and country, between the economy and the environment, and between western scientific and indigenous knowledge systems begin to flourish, with stakeholders in government, business, research, and civil society working together to deepen these connections and build a shared vision for a nature-positive society and economy, guided by science-based targets aligned with the SDGs.  Governments at local, regional, state, territory and national levels align under this national vision in collaboration with partners, to improve the coordination of policies and programs designed to tackle major threats to both the terrestrial and marine environments. These include management of protected areas, protection of heritage, and measures to protect threatened species and ecological communities, and to promote their longer-term recovery. Australia’s strategies and investments in biodiversity conservation begin to align with the scale of the challenge to turnaround key trends of decline in ecosystems. Transforming Australia’s economy and society more broadly are central to achieving a nature-positive goal, whereby critical biodiversity targets and indicators show a net-positive outcome. Here, the successful delivery of the other transformation pathways will have a strong influence on transformation of the environmental commons, particularly food systems (**T3**), dominant patterns of production and consumption (**T2**), energy decarbonisation (**T4**), and urban systems (**T5**).  In line with this narrative, specific quantitative interventions (**Supplementary Table 3**) to support this transformation include investment in reforestation, additional terrestrial protected area expenditure, and additional marine park expenditure. | ^[48, 51, 61-64]^ |

## Supplementary Table 3. Pathway Policy Settings and Assumptions – BBS and STP Policy Levers

| **Interventions** | **Build Back the Same (BBS)** | **Six Transformations Pathway (STP)** | **Source/Notes** |
| --- | --- | --- | --- |
| **T1. Wellbeing and Capabilities** |  |  |  |
| Additional education expenditure as % GDP | Return to Pre-COVID  2020: 0.11  2021: 0.09  2022: 0  2023-2030: 0 | Increase to 2030  2020: 0.11  2021: 0.5  2022: 0.3  2023-2030: 0.3 | Increased access to vocational training through Job Maker Program and additional research funding during COVID. STP assumes further investment in education for job retention. ABS government finance statistics and Australian Government budget ^[18, 65, 66]^ |
| Additional health expenditure as % GDP | Return to Pre-COVID  2020: 0.695  2021: 0.04  2022: 0  2023-2030: 0 | Increase to 2030  2020: 0.695  2021: 0.5  2022: 0.3  2023-2030: 0.3 | Increased expenditure on vaccine availability, rollout and preparing hospitals for pandemic. STP with ongoing investment into health industries, particularly as a result of age care inquest. Government education expenditure from ABS government finance statistics and Australian Government budget ^[18, 65, 66]^ and benchmarked on OECD data for general government expenditure ^[67]^ |
| Additional adaptation expenditure as % GDP | Baseline investment to 2050  2020: 0.008  2021: 0.01  2022: 0.01  2023-2030: 0.01  2050: 0.01 | Increased investment to 2050  2020: 0.008  2021: 0.1  2022: 0.1  2023-2030: 0.1  2050: 0.1 | STP assumes increased investment. Adaptation costs estimates ^[15-17]^. |
| **T2. Sustainable and Just Economies** | | | |
| Additional expenditure on subsidies and transfers as % GDP | Some continued allocation to 2030  2020: 9.4  2021: 0.605  2022: 0.1172  2023-2030: 0.115 | Large increase post-COVID to 2030  2020: 9.4  2021: 1  2022: 0.5  2023-2030: 0.5 | Including both government fiscal policy (job Keeper, job Seeker & Covid-19 supplements). Some continued injection through job seeker supplements over forward years. Government general expenditure data from ABS government finance statistics and Australian Government budget ^[18, 65, 66]^ and benchmarked on OECD social transfers data ^[68]^ |
| Relative fiscal pressure by percentile future | Existing trend  0.8,0.9,1,1.4,0.4 | Increased for high/ decreased for low-income earners  0.8,0.9,1,1.2,1 | STP assumes a shift in tax burden to higher income earner quintiles and abandoning Stage 3 tax cuts. Parameters based on i*SDG* model settings ^[1]^ calibrated on income distribution and poverty data from ABS household income and wealth data ^[69]^ and HILDA survey data ^[70]^. Adjusted for additional fiscal pressure on high/low income quintiles. |
| Target subsidies and transfers distribution | Existing trend | More targeted at lower incomes  [(0,0)-(100,10)],(1,1.2),(100,0.9) | Parameterised based on iSDG model settings ^[1]^ with adjustments in favour of low-income earners for the STP trajectory |
| Gender employment gap target effect | No change (0.08) | Improved gender equality from 2021 (0.06) | Improvement in STP based on policy proposal within Labor policies which would include an enhanced childcare system, with coverage reaching more people: 6.2 billion over 4 years cost estimate ^[66]^. |
| Additional tax rate on international trade as % GDP | No change | Increased tax rate  +0.13% | STP– Equivalent to increase in tax by ~15%.  International trade tax rates based on IMF data for Australia 1990-2018 ^[71]^. Low-medium parameters estimated based on BAU and timeseries data. |
| Tax rate on income and profits as % GDP | Decreased  2020: -0.43  2021: -1.6  2022: -0.75  2023-2030: -0.75  2050: -0.75 | Increased tax rate  2020: -0.43  2021: 2.6  2022: 2.6  2023-2030: 2.6  2050: 2.6 | Government stage 1 and 2 tax cuts and write offs reduce income. STP – government drops stage 3 tax cuts and increases revenue to cover additional spending. Increase is equivalent to a 15% increase on the existing tax revenue, this would equate to a total average tax rate of 27.3% which is similar to Norway (OECD). Based on IMF data ^[71]^. |
| Additional tax rate on consumption (GST) | No Change | Increased tax rate  2020: 0  2021: 0.77  2022: 0.77  2023-2030: 0.77  2050: 0.77 | For STP, increase to 11.5% GST. Outcome of tax reform and Government Tax Summit. Based on IMF data ^[71]^. |
| Additional infrastructure (paved road and railway) expenditure as % GDP | Continued investment to 2030 (mainly roads)  2020: 0.145; 0.066  2021: 0.23; 0.1  2022: 0.24; 0.11  2023-2030: 0.23; 0.1 | Increased investment to 2030  2020: 0.145; 0.066  2021: 0.2; 0.4  2022: 0.2; 0.4  2023-2030: 0.2; 0.4 | Australian Government Building Our Future – additional infrastructure investment.  ABS Government Finance Statistics ^[18]^ and road/rail expenditure statistics ^[72]^ general government expenditure on transportation infrastructure. Parameterised based on historic data and high estimates for road or rail for STP. |
| Investment in green manufacturing transition ($) | None | Additional investment of $20 billion to 2030 | STP assumption based on Beyond Zero Emissions report $20 billion facility to undertake large scale investment in grid infrastructure for green industry ^[35]^. Sectors: green aluminium, green steel, green hydrogen. Studies estimate green manufacturing production at an additional $180 bn per annum ^[73]^. |
| Final target material consumption efficiency improvement in 2030 and 2050 (%) | None | Increased targets  2030: 0.12 (12%)  2050: 0.22 (22%) | STP assumes improvement in material efficiency through R&D in industry regulation. Material efficiency target estimated based on CSIRO material flows data for Australia 1990-2017 ^[74]^. |
| **T3. Sustainable Food Systems & Healthy Nutrition** | | | |
| Additional agriculture water efficiency expenditure as % GDP | Some continued investment to 2030  2020: 0.018  2021: 0.053  2022: 0.03  2023-2030: 0.03 | Increased investment to 2030  2020: 0.018  2021: 0.07  2022: 0.07  2023-2030: 0.06 | Water reforms in Murray Darling Basin as well as research in water industry. Water efficiency expenditure estimated based on Hickey, Hoogers ^[75]^, Government of Victoria ^[76]^ and FAOSTAT database ^[77]^. Australian Government Water Fund allocation of $2.6 billion over 6 years (AUD2015); 4.3ML/ha average water usage all crops ^[75]^. Cost of enhanced irrigation  = $2,100/ha/pa average ^[76]^. Total irrigated area = 23m ha ^[77]^  20% = $9.66bn |
| Additional sustainable agriculture training expenditure as % GDP | None | Increased investment to 2030  2020: 0.045  2021: 0.05  2022-2030: 0.07 | STP assumes investment in regenerative agriculture extension services – Farms of the Future. Agriculture training expenditure estimated based on ABS Labour Force data for Australia 1990-2016 ^[78]^ on agricultural employment and estimate of $5,000 USD 2010 cost per farmer. |
| Target emissions reduction from livestock and crops by 2050 (%) | Moderate Reductions in 2030 and 2050  2030: 0.07 (7%); 0.05 (5%)  2050: 0.15 (15%); 0.15 (15%) | Rapid Reductions by 2030 and 2050  2030: 0.4 (40%); 0.1(10%)  2050: 0.83 (83%); 0.27 (27%) | Estimates for reduction in agriculture emissions based on mitigation pathways from ClimateWorks Australia ^[48]^ - medium and high ambition scenarios. |
| **T4. Energy Decarbonisation with Universal Access** | | | |
| Additional large-scale hydropower capacity expenditure as % GDP | None | Additional investment to 2025  2025: 0.1  2030: 0 | Based on CSIRO and ClimateWorks ^[48, 79, 80]^ |
| Additional small- and large-scale photovoltaic capacity expenditure as % GDP | None | Additional investment to 2030  2020: 0.05; 0.015  2021: 0.4; 0.025  2022: 0.35; 0.025  2023-2030: 0.35; 0.02 | STP - Additional investment in supporting infrastructure; grid upgrades. Based on CSIRO and ClimateWorks ^[48, 79, 80]^ |
| Additional expenditure on wind energy as % GDP | None | Additional investment to 2030  2021: 0.02  2030:0.05 | STP additional expenditure based on ClimateWorks Australia ^[48]^ |
| Additional industry energy efficiency expenditure as % GDP | Continued investment to 2030  2020: 0.007  2021: 0.009  2022: 0.01  2023-2030: 0.01 | Increased investment to 2030  2020: 0.007  2021: 0.2  2022: 0.2  2023-2030: 0.2 | Securing energy policies included in the Job Maker program for energy efficiency. For STP, calibrated on reduction targets based on ClimateWorks and Australia Energy Efficiency Strategy ^[48, 81]^ |
| Fuel switch from gas and oil/coal used in industry (%) | Modest fuel switching by 2050  2050: 0.1(10%); 0.4 (40%) | Strong fuel switching by 2050  2050: 0.3 (30%); 0.8 (80%) | STP assumes accelerated industry fuel switching to electrification and away from fossil fuels over the long-term. Estimated based on ^[48]^ |
| Carbon capture and storage reduction target for industry fugitive emissions by 2050 (%) | Some CCS for fugitive emissions by 2050  2050: 0.1 (10%) | Increased CCS for fugitive emissions by 2050  2050: 0.6 (60%) | STP improved CCS from industry based on ClimateWorks (2020) |
| Additional gas capture at waste disposal sites by 2050 (%) | Some gas capture from waste by 2050  2050: 0.1 (10%) | Increased gas capture from waste by 2050  2050: 0.6 (60%) | STP assumes increased gas capture from waste disposal sites, based on ClimateWorks (2020) |
| Target reduction in industrial process emissions by 2050 (%) | Some reduction by 2050:  2050: 0.1 (10%) | Increased reduction by 2050  2050: 0.5 (50%) | STP assumes increased reduction of industrial process emissions based on ClimateWorks (2020) |
| Deliberate phase out of fossil fuel capacity generators | None | Accelerated phase out of fossil fuel generators | STP assumes no new coal and gas generation and accelerated phase out of coal and gas generation, increasing pace after 2030, based on ClimateWorks (2020). |
| **T5. Urban and Peri-Urban Development** | | | |
| Additional vehicle efficiency expenditure as % GDP | None | Increased investment to 2030  2021: 0.5  2022: 0.5  2023-2030: 0.5 | STP assumes increased investment in vehicle efficiency. Additional expenditure parameterised based on Commonwealth of Australia ^[82]^ data on fuel efficiency. |
| Additional energy efficiency expenditure as % GDP – services and households | None | Increased investment to 2030  2021: 0.2  2022: 0.2  2023-2030: 0.2 | STP assumes increased investment in households and commercial buildings. Targets based on ClimateWorks and Australia Energy Efficiency Strategy ^[48, 81]^ |
| Fuel switch commercial buildings by 2030 and 2050 (%) | Baseline electrification by 2050  2050: 0.3 (30%) | Accelerated electrification by 2050  2050: 1 (100%) | STP assumes accelerated electrification of commercial buildings. Targets and switching based on ClimateWorks and ASBEC ^[48, 83]^ and previous modelling for Australia ^[84]^ |
| Fuel switch residential buildings by 2030 and 2050 (%) | Baseline electrification by 2050  2050: 0.3 (30%) | Accelerated electrification by 2050  2050: 1 (100%) | STP assumes accelerated electrification of residential buildings. Targets and switching based on ClimateWorks and ASBEC ^[48, 83]^ and previous modelling for Australia ^[84]^ |
| Target share of timber buildings by 2050 | None | Mandated target for 2050  2050: 0.3 (30%) | STP assumes a mandated target for timber buildings in 2050, based on national modelling for Australia ^[84]^. Building material intensities based on Skullestad, Bohne ^[85]^ and Allen, Oldfield ^[84]^ |
| Target improvement in timber resource recovery and reuse by 2050 | No increase  Baseline = 0.42 (42%) | Target increased by 2050  2050: 0.8 (80%) | STP assumes increased timber resource recovery and reuse by 2050. Estimates of average carbon sequestration and average end of life emissions based on national data ^[86]^. |
| Target reduction in per capita waste generation by 2030 and 2050 | Some baseline improvement  2030: 0.05 (5%)  2050: 0.1 (10%) | Increased improvement  2030: 0.15 (15%)  2050: 0.3 (30%) | STP assumes greater reduction in per capita waste generation based on the National Waste Policy Action Plan ^[87]^ |
| Target reduction in non-recycled municipal waste due to improved waste management by 2030 and 2050 (%) | Some baseline improvement  2030: 0.1 (10%)  2050: 0.2 (20%) | Increased improvement  2030: 0.35 (35%)  2050: 0.6 (60%) | Based on the National Waste Policy Action Plan ^[87]^ with improved resource recovery from non-recycled solid waste. Assumes increased improvement under STP – 15% organic waste; 20% recycled content (maximum expected reduction 35% by 2030). |
| Target share of new vehicles electric by 2030, 2040, 2050 (%) – by vehicle type (passenger, commercial, cycle, articulated trucks, other trucks, buses) | 2030: 0.1, 0.1, 0.1, 0.05, 0.1, 0.1  2040: 0.4, 0.4, 0.4, 0.3, 0.4, 0.4  2050: 0.65, 0.65, 0.65, 0.65, 0.65, 0.65 | 2030: 0.4, 0.3, 0.3, 0.2, 0.3, 0.4  2040: 0.9, 0.9, 0.9,0.7,0.9,0.9  2050: 1, 1, 1, 0.9, 1, 1 | STP assumes accelerated shift to BEVs in new vehicle sales for all types. Baseline based on AEMO projections ^[88, 89]^. STP based on ^[48, 90, 91]^ |
| Forced phase out of internal combustion vehicles | None. Standard vehicle phase out (20, 22, 20, 24, 22, 20) | Accelerated phase out to 2045 (10, 10, 10, 10, 10, 10)  Complete phase out from 2045 to 2050 (1, 1, 1, 1, 1, 1) | STP assumes an accelerated phase out and then complete phase out from 2045, based on ^[90, 92, 93]^. |
| Required investment in electric vehicle subsidies and charging infrastructure (%GDP) | Modest expenditure to 2050  2021: 0.001  2030: 0.013  2050: 0.021 | Increased expenditure to 2050  2021: 0.001  2030: 0.017  2050: 0.036 | Modelled costs based on BEV uptake calibrated on Broadbent, Allen ^[91]^ |
| **T6. Global Environmental Commons** | | | |
| Additional reforestation expenditure as % GDP | None | Increased investments to 2030  2021: 0.05  2022: 0.04  2023-2030: 0.02 | STP assumes increased investment in carbon forestry and reforestation. Reforestation expenditure estimated based on Summers, Bryan ^[94]^, Landcare expenditure data ^[95]^ and ^[48]^ |
| Additional marine park protected areas expenditure as % GDP | None | Increased investments to 2050  2021: 0.02  2022: 0.02  2023-2030: 0.02  2050: 0.02 | STP assumes increased investment in marine protected areas. Expenditure for marine protected areas estimated based on Balmford, Gravestock ^[96]^, Ban, Adams ^[97]^ |
| Additional terrestrial protected areas expenditure as % GDP | None | Increased investments to 2050  2021: 0.035  2022: 0.035  2023-2030: 0.035  2050: 0.035 | STP assumes increased investment in terrestrial protected areas. Expenditure for terrestrial protected areas estimated based on Adams, Segan ^[98]^, James, Green ^[99]^ and ABS government expenditure data on environmental heritage ^[100]^ |

## Supplementary Table 4. Evaluation framework of SDG Targets and Indicators (2030) and Transformation Targets (2050)

| **Goal** | **Target (52)** | **T^#^ (67)** | **D^##^** | **Indicators (80)** | **Indicator in *iSDG-Australia* (80)** | **Baseline Value (~2015)** | **Target value 2030** | **Target value 2050** | **Target source** |
| --- | --- | --- | --- | --- | --- | --- | --- | --- | --- |
| 1 | 1.2 By 2030, reduce at least by half the proportion of men, women and children of all ages living in poverty in all its dimensions according to national definitions | 2 | E | 1.2.1 | Proportion of population below national poverty line (% population) | 0.12 | 0.06 | 0.03 | SDG Target 1.2 is to halve baseline value ^[101]^ by 2030. Target set to halve again by 2050 (-75%) |
|  | 1.5 By 2030, build the resilience of the poor and those in vulnerable situations and reduce their exposure and vulnerability to climate-related extreme events and other economic, social and environmental shocks and disasters | 1 | S | 1.5.1 | Mortality due to disasters five-year average (% population) | 0.0000021  2.1e-6 | 1.05e-6 | No change | Sendai Framework DRR targets for 2030 are to ‘substantially reduce’ ^[102]^; target set as 50% improvement on baseline value by 2030. |
|  |  |  |  |  |  |  |  |  |  |
| 2 | 2.1 By 2030, end hunger and ensure access by all people, in particular the poor and people in vulnerable situations, including infants, to safe, nutritious and sufficient food all year round | 3 | S | 2.1.1 | Prevalence of undernourishment | 0.025 | 0.0125 | No change | SDG Target 2.1 is to ‘end hunger’ ^[101]^; target set as 50% improvement on baseline by 2030. |
|  | 2.1 | 3 | S | 2.1.2.ALT | Prevalence of food insecurity: Proportion of population below the food poverty line | 0.056 | 0.028 | No change | SDG Target 2.1 is to end hunger ^[101]^; target set as 50% improvement on baseline by 2030 |
|  | 2.2 By 2030, end all forms of malnutrition, including achieving, by 2025, the internationally agreed targets on stunting and wasting in children under 5 years of age… | 3 | S | 2.2.1 | Prevalence of stunting | 0 | 0 | No change | SDG Target 2.2 already achieved for Australia. Maintain performance. |
|  | 2.2 | 3 | S | 2.2.2 | Prevalence of malnutrition (%) | 0 | 0 | No change | SDG Target 2.2 already achieved for Australia. Maintain performance. |
|  | 2.3 By 2030, double the agricultural productivity and incomes of small-scale food producers, in particular women, indigenous peoples, family farmers, pastoralists and fishers… | 3 | E | 2.3.1 | Total agriculture production in tons per labor unit (t/person) | 342.7 | 582.6 | No change | SDG Target 2.3 is to double productivity of small-scale farmers compared to baseline value ^[101]^. Historic rate of improvement = ~110% since 1990. Target set at 70% improvement on baseline by 2030. |
|  | 2.3 |  | E | 2.3.1Alt | Cereal yield (t/ha) | 2.2 | 2.8 | No change | SDG Target 2.3 is to double productivity of small-scale farmers compared to baseline value ^[101]^. Historic rate of improvement = ~30% since 1990. Target set as 30% improvement on baseline by 2030. |
|  | 2.4 By 2030, ensure sustainable food production systems and implement resilient agricultural practices that increase productivity and production… | 3 | V | 2.4.1 | Proportion of harvested area sustainably managed (organic) (%) | 0.2087 | 0.5 | 0.8 | SDG Target is vague. Target set as 50% of harvested area by 2030, calibrated off historic data from FAO (increase of 1% to 25% in 10 years; historic peak of 27%) ^[77]^. Increased to 80% by 2050. |
|  |  | 3 | V | 2.4.1alt | Fertilizer consumption per capita (kg N/capita/year) | 53.7 | 26.9 | 13.4 | 50% reduction by 2030, and 75% reduction by 2050 to bring closer to per capita planetary boundary of 8.9 kg ^[103]^. |
|  |  |  |  |  |  |  |  |  |  |
| 3 | 3.1 By 2030, reduce the global maternal mortality ratio to less than 70 per 100,000 live births | 1 | S | 3.1.1 | Maternal mortality ratio (per 100,000 live births) | 6 | 6 | No change | Target already achieved for Australia. Maintain performance. |
|  | 3.2 By 2030, end preventable deaths of newborns and children under 5 years of age, with all countries aiming to reduce neonatal mortality to at least as low as 12 per 1,000 live births and under‑5 mortality to at least as low as 25 per 1,000 live births | 1 | S | 3.2.1 | Under five mortality rate (per 1,000 live births) | 5 | 5 | No change | SDG Target 3.2 already achieved for Australia. Maintain performance. |
|  | 3.2 | 1 | S | 3.2.2 | Neonatal mortality rate (per 1,000 live births) | 2.3 | 2.3 | No change | SDG Target 3.2 already achieved for Australia. Maintain performance. |
|  | 3.4 By 2030, reduce by one third premature mortality from non-communicable diseases through prevention and treatment and promote mental health and well-being | 1 | S | 3.4.1 | Cardiovascular neoplasm diabetes and respiratory mortality (per 1,000 population) | All: 0.00417 | 0.00278 | No change | SDG Target 3.4 is to reduce by 1/3 on baseline value by 2030 ^[101]^ |
|  | 3.6 By 2020, halve the number of global deaths and injuries from road traffic accidents | 1 | S | 3.6.1 | Total road fatalities (persons) | 1471 | 1176 | No change | SDG Target 3.6 is to halve global baseline value ^[101]^. Historic rate of improvement = ~16% since 1990. Target set as 20% reduction by 2030. |
|  | 3.7 By 2030, ensure universal access to sexual and reproductive health-care services… | 1 | S | 3.7.2 | Adolescent birth rate (per 1,000) | 15.8 | 11.9 | No change | Target set as 25% improvement on baseline value by 2030; calibrated from historic data and BAU projection ^[19, 104]^ |
|  | 3.8 Achieve universal health coverage | 1 | S | 3.8.1 | Average access to basic health care (%) | 0.99 | 0.99 | No change | SDG Target 3.8 already achieved for Australia. Maintain performance. |
|  |  |  |  |  |  |  |  |  |  |
| 4 | 4.1 By 2030, ensure that all girls and boys complete free, equitable and quality primary and secondary education leading to relevant and effective learning outcomes | 1 | S | 4.1.1 | Proportion of population age 20 to 24 that has completed secondary school (%) | 0.86 | 0.9 | 0.92 | SDG Target 4.1 is ‘universal completion’ ^[101]^. Target level of 0.9 (90%) by 2030 set based on historic data and BAU projection ^[19, 105]^. Increase to 92% by 2050. |
|  | 4.3 By 2030, ensure equal access for all women and men to affordable and quality technical, vocational and tertiary education, including university | 1 | S | 4.3.1 | Proportion of population age 20 to 29 that has enrolled in tertiary education (%) | 0.5 | 0.75 | No change | Target set as 50% improvement on baseline value; calibrated off historic data and BAU projection ^[19, 105]^. |
|  | 4.3 | 1 | S | 4.3.1.ALT | Gender parity ratio in tertiary education (women proportion of total tertiary graduates) | 0.53 | ~0.5 | No change | SDG Target 4.3 is for ‘equal access’ or gender parity ^[101]^. Target set as maintain at ~50% (+/-0.03%). |
|  | 4.5 By 2030, eliminate gender disparities in education and ensure equal access to all levels of education and vocational training for the vulnerable, including persons with disabilities, indigenous peoples and children in vulnerable situations | 1 | S | 4.5.1 | Adult literacy gender gap ratio | 0.01 | 0.01 | No change | SDG Target 4.5 is to ‘eliminate gender disparity’ ^[101]^. Target set as maintain at ~0% (+/-0.03%). |
|  | 4.6 By 2030, ensure that all youth and a substantial proportion of adults, both men and women, achieve literacy and numeracy | 1 | S | 4.6.1 | Average adult literacy rate (%) | 0.97 | 0.97 | No change | SDG Target 4.6 is a ‘substantial proportion’ of adults ’ ^[101]^. Target already achieved for Australia. Maintain performance. |
|  | 4.6 | 1 | S | 4.6.1alt | Total average years of schooling | 11.73 | 12.3 | 13 | Target calibrated from historic data and BAU projection and set at 12.3 by 2030 and 13 by 2050 ^[106]^. |
|  |  |  |  |  |  |  |  |  |  |
| 5 | 5.5 Ensure women’s full and effective participation and equal opportunities for leadership at all levels of decision-making in political, economic and public life | 2 | S | 5.5.1 | Proportion of female legislators senior officials and managers (%) | 0.37 | 0.5 | No change | SDG Target 5.5 is for ‘equal opportunity’ ^[101]^. Target set as gender parity ~0.5. |
|  | 5.5 | 2 | E | 5.5.1.ALT | Employment to population ratio (female/male parity) | 0.839 | 0.95-1.05 | No change | SDG Target 5.5 is for ‘equal opportunity’ ^[101]^. Target set as gender parity = ~1. |
|  | 5.6 Ensure universal access to sexual and reproductive health and reproductive rights… | 1 | S | 5.6.1 | Contraceptive prevalence rate (%) | 0.72 | 0.72 | No change | Target set as ‘maintain’. Baseline value is comparably high compared with other developed countries (OECD average = 70.5) ^[19]^. |
|  |  |  |  |  |  |  |  |  |  |
| 6 | 6.1 By 2030, achieve universal and equitable access to safe and affordable drinking water for all | 5 | S | 6.1.1 | Average access to improved water source (%) | 1 | 1 | No change | SDG Target 6.1 already achieved for Australia. Maintain performance. |
|  | 6.2 By 2030, achieve access to adequate and equitable sanitation and hygiene for all | 5 | S | 6.2.1 | Average access to improved sanitation facility (%) | 1 | 1 | No change | SDG Target 6.2 already achieved for Australia. Maintain performance. |
|  | 6.4 By 2030, substantially increase water-use efficiency across all sectors and ensure sustainable withdrawals and supply of freshwater… | 2 | V | 6.4.1 | Total water withdrawal per unit of GDP (m3/$real GDP) | 0.011 | 0.008 | 0.0055 | SDG Target 6.4 is for a ‘substantial increase’ ^[101]^. Target set as 30% improvement on baseline value by 2030; calibrated from historic trend data, BAU projection and global trend and outlook ^[14, 107, 108]^.^[109]^. Increased to 40% reduction by 2050. |
|  | 6.4 | 5 | V | 6.4.1.ALT1 | Water consumption per capita (m3/capita) | 761.2 | 571 | No change | SDG Target 6.4 is for a ‘substantial increase’ ^[101]^. Target set as 25% improvement on baseline by 2030; calibrated off historic trend and timeseries data ^[107, 110]^. By 2050, maintain below planetary boundary of 574 m3 per capita per year ^[103]^ |
|  | 6.4 | 3 | V | 6.4.1.ALT2 | Agricultural consumption of water and gross value added by irrigated agricultural production (L/$ real GVIAP) | 0.52 | 0.39 | 0.34 | SDG Target 6.4 is for a ‘substantial increase’ ^[101]^. Target set as 25% improvement on baseline by 2030; calibrated off historic trend and timeseries data ^[14, 107, 110]^. Increase to 35% by 2050. |
|  | 6.4 | 6 | V | 6.4.2 | Water resources vulnerability index (%) | 0.037 | <=0.05 | No change | SDG Target 6.4 is for ‘sustainable withdrawal and supply’. Index score is low for Australia. A country is considered water scarce if annual withdrawals are between 20 and 40% of annual supply, and severely water scarce if withdrawals exceed 40% (Raskin et al., 1997).Target set as maintain at 0.05 or below; calibrated based on historic timeseries data and BAU projection ^[14, 107]^. |
|  |  |  |  |  |  |  |  |  |  |
| 7 | 7.1 By 2030, ensure universal access to affordable, reliable and modern energy services | 4 | S | 7.1.1 | Percentage of population with access to electricity (%) | 100 | 100 | No Change | SDG Target 7.1 already achieved for Australia. Maintain performance. |
|  | 7.2 By 2030, increase substantially the share of renewable energy in the global energy mix | 4 | V | 7.2.1 | Renewable share in total final energy consumption (%) | 0.094 | 0.3 | 0.6 | Target set based on National Sustainable Development Council (NSDC) target of 30% by 2030 ^[31, 111]^. Increase to 60% by 2050 ^[48]^. |
|  | 7.2 | 4 | V | 7.2.1.ALT | Renewable share in electricity (%) | 0.13 | 0.55 | 0.95 | Target set based on National Sustainable Development Council (NSDC) target of 55% by 2030 ^[31]^. Increase to at least 95% by 2050 ^[48]^ |
|  | 7.3 By 2030, double the global rate of improvement in energy efficiency | 2 | V | 7.3.1 | Energy intensity level of primary energy (MJ/USD11) | 5.06 | 3.04 | 2.02 | SDG Target 7.3 is to double the rate of improvement; calibrated from historic timeseries data (equates to target of 3.7%pa; or ~50% total) ^[112, 113]^. COAG NEPP target is 40% increase in energy productivity 2015 to 2030 (~2.67%pa) ^[114]^. Target set at reduce by 40% by 2050, and 60% by 2050. |
|  |  |  |  |  |  |  |  |  |  |
| 8 | 8.1 Sustain per capita economic growth in accordance with national circumstances… |  | E | 8.1.1 | Real pc GDP growth rate 5-year average (% pa) | 0.007 | 0.005 | No change | SDG Target 8.1 is to ‘sustain’ per capita growth. Target set at 0.5% pa; calibrated from timeseries data and BAU projection ^[109]^. |
|  | 8.1 | 2 | E | 8.1.1.ALT2 | Per capita real disposable income (real $/person/year) | 55,908 | 61,500 | No change | Target set as 10% improvement on baseline value by 2030; calibrated from timeseries data and BAU projection ^[115]^. |
|  | 8.2 Achieve higher levels of economic productivity through diversification, technological upgrading and innovation, including through a focus on high-value added and labour-intensive sectors | 2 | E | 8.2.1 | GDP per employed person growth rate (%) | 0.01 | 0.01 | No change | SDG Target 8.2 is higher productivity. Target set as 1% pa; calibrated from timeseries data and BAU projections ^[78, 109]^. |
|  | 8.4 Improve progressively, through 2030, global resource efficiency in consumption and production and endeavor to decouple economic growth from environmental degradation | 2 | V | 8.4.1c | Material footprint per unit of output (kg/USD11) | 0.82 | 0.58 | 0.041 | Target set at 30% total improvement on baseline by 2030; calibrated from global data and BAU projection ^[74]^. Increased to 50% reduction by 2050. |
|  | 8.4 | 2 | V | 8.4.2c | Domestic material consumption per unit of output (kg/USD11) | 0.77 | 0.46 | 0.031 | Target set at 40% improvement on baseline by 2030; calibrated from global data and BAU projection ^[74]^, and IRP target 36-54% by 2050 ^[116]^. Increased to 60% by 2050. |
|  | 8.5 By 2030, achieve full and productive employment and decent work for all women and men, including for young people and persons with disabilities, and equal pay for work of equal value | 2 | E | 8.5.2 | Unemployment rate (%) | 0.06 | 0.05 | No change | SDG Target 8.5 is ‘full employment’. Target set at >95% employment (i.e. approximately ‘natural rate’ of unemployment – long-term average 5.3%) ^[78]^. |
|  | 8.5 | 2 | E | 8.5.2.ALT | Average employment to adult population ratio | 61.8 | 65.9 | 69 | Target calibrated from timeseries data ^[78]^.and NSDC target 0f 65.9 by 2030 ^[111]^. Increase to 69 by 2050. |
|  |  |  |  |  |  |  |  |  |  |
| 9 | 9.1 Develop quality, reliable, sustainable and resilient infrastructure, including regional and transborder infrastructure, to support economic development and human well-being, with a focus on affordable and equitable access for all | 2 | E | 9.1.1 | Rural Access Index (%) | 0.93 | 0.98 | No change | Calibrated from based on iSDG model calibration for Australia. Target set as universal access (=>98%). |
| 9 | 9.1 |  | E | 9.1.1.ALT | Public infrastructure investment (transportation expenditure as % GDP) | 0.018 | 0.021 | No change | Calibrated from historic data; max 0.22 in 1990s) ^[72, 117-119]^ and NSDC target of 2.2% by 2030. |
|  | 9.2 Promote inclusive and sustainable industrialization and, by 2030, significantly raise industry’s share of employment and gross domestic product, in line with national circumstances, and double its share in least developed countries | 2 | E | 9.2.1a | Manufacturing value added as % GDP fc (%) | 0.07 | 0.077 | No change | SDG Target 9.2 is to increase share. SDG target set at 10% improvement on baseline; calibrated from timeseries data (max 0.14) ^[109, 120]^. |
|  | 9.2 | 2 | E | 9.2.1b | PC manufacturing value added (real $/person/year) | 4,667 | 4478 | No change | SDG Target 9.2 is to increase share. Target set as 10% improvement on baseline; calibrated from timeseries data (max 5,700) ^[109, 110, 120]^. |
|  | 9.2 |  | E | 9.2.2 | Manufacturing employment as share of total employment (%) | 0.09 | 0.10 | No change | SDG Target 9.2 is to increase share. Target set as 10% improvement on baseline; calibrated from timeseries data (max 0.14) ^[78, 120]^. |
|  | 9.4 By 2030, upgrade infrastructure and retrofit industries to make them sustainable, with increased resource-use efficiency and greater adoption of clean and environmentally sound technologies and industrial processes, with all countries taking action in accordance with their respective capabilities | 2 | V | 9.4.1 | CO2 emissions per unit of value added (kg CO2-e/USD 2011) | 0.31 | 0.19 | 0.03 | SDG Target 9.4 is to increase efficiency. Target set as 40% improvement on baseline value by 2030; calibrated from timeseries data and global data (OECD average = 0.21) ^[109, 121, 122]^. Increased to 90% reduction by 2050. |
|  |  |  |  |  |  |  |  |  |  |
| 10 | 10.1 By 2030, progressively achieve and sustain income growth of the bottom 40 per cent of the population at a rate higher than the national average | 2 | E | 10.1.1 | Bottom 40 percent income growth to average income growth gap | -0.002 | 0.003 | No change | SDG Target 10.1 is to achieve and sustain growth. Target set as equal to or greater than 0.3% growth pa; calibrated from timeseries data ^[69]^. |
|  | 10.1 | 2 | E | 10.1.1.ALT1 | Gini coefficient income | 0.33 | 0.29 | No change | Target set based on SDG Index green threshold ^[123, 124]^ and calibrated from timeseries data ^[69, 70]^. |
|  | 10.1 | 2 | E | 10.1.1.ALT2 | Palma ratio income (top 10%/bottom 40%) | 1.25 | 0.99 | No change | Target set based on SDG Index green threshold ^[123, 124]^ and calibrated from timeseries data ^[69, 70]^. |
|  | 10.4 Adopt policies, especially fiscal, wage and social protection policies, and progressively achieve greater equality | 2 | E | 10.4.1 | Average labor share of GDP, comprising wages and social protection transfers | 0.69 | 0.76 | No change | Target set as 10% improvement on baseline value; calibrated from timeseries data and BAU projection ^[115]^. |
|  |  |  |  |  |  |  |  |  |  |
| 11 | 11.2 By 2030, provide access to safe, affordable, accessible and sustainable transport systems for all, improving road safety, notably by expanding public transport, with special attention to the needs of those in vulnerable situations, women, children, persons with disabilities and older persons | 5 | V | 11.2.1.ALT1 | Share of battery electric vehicles (BEVs) in total vehicle (%) | 0.00002 | 0.2 | 0.90 | Target set as 20% of the total number of vehicles to be BEVs by 2030. Calibrated off data on vehicles and BEVs ^[92, 93, 125]^ and BAU projection. Increased to 90% share by 2050. |
|  | 11.2 | 5 | V | 11.2.1ALT2 | Total greenhouse gas emissions from road transport sector (Mt CO2-eq) | 73.9 | 65.032 | 7.4 | 12% reduction by 2030 and 95% reduction by 2050 ^[48]^ |
|  | 11.5 By 2030, significantly reduce the number of deaths and the number of people affected and substantially decrease the direct economic losses relative to global gross domestic product caused by disasters, including water-related disasters, with a focus on protecting the poor and people in vulnerable situations | 5 | E | 11.5.2 | Economic damage due to natural disasters as share of GDP five-year average (% GDP) | 0.0023 | 0.0013 | No change | Sendai Framework DRR targets are to ‘substantially reduce’ ^[102]^; target set as 50% improvement on baseline value. |
|  | 11.6 By 2030, reduce the adverse per capita environmental impact of cities, including by paying special attention to air quality and municipal and other waste management | 5 | V | 11.6.1 | Proportion of urban waste collected and disposed (%) | 1 | 1 | No change | SDG Target 11.6 already achieved for Australia. Maintain performance. |
|  | 11.6 | 5 | V | 11.6.2 | Proportion population exposed to PM2.5 levels exceeding WHO guideline | 0.34 | 0.24 | No change | Guideline for exposure of 10 μg/m^3 [126]^. SDG Target 11.6 is to reduce per capita environmental impacts. Target set at 30% improvement on baseline. |
|  | 11.6 | 5 | V | 11.6.NEW | Total operational and embodied GHG emissions in the built environment (Mt CO2-eq) | 117.57 | 42.3 | 0 | 64% reduction by 2030 and 100% by 2050 ^[48, 84]^ |
|  |  |  |  |  |  |  |  |  |  |
| 12 | 12.2 By 2030, achieve the sustainable management and efficient use of natural resources | 6 | V | 12.2.1 | PC material footprint (t/person/year) | 42.1 | 25.3 | 21.1 | Target calibrated from global data and BAU projection ^[74]^ and NSDC 2030 target value of 25.3 based on OECD average ^[111]^. Total reduction of 50% by 2050. |
|  | 12.2 | 6 | V | 12.2.2 | PC domestic material consumption (t/person/year) | 39.4 | 27.6 | 15.8 | Target set at 30% total improvement on baseline by 2030; calibrated from global data and BAU projection ^[74]^. Reduce by 60% by 2050 to bring closer to per capita planetary boundary of 7.2t. |
|  | 12.5 By 2030, substantially reduce waste generation through prevention, reduction, recycling and reuse | 5 | V | 12.5.1alt | Non-recycled municipal solid waste per capita (kg) | 585.1 | <365 | -55% | SDG Index green threshold ^[127]^ based on top performing countries and OECD data ^[111]^. National Waste Action Plan (2020) has a target to reduce by around 35% by 2030. Reduce by 55% by 2050. |
|  |  |  |  |  |  |  |  |  |  |
| 13 | 13.1 Strengthen resilience and adaptive capacity to climate-related hazards and natural disasters in all countries | 1 | S | 13.1.2 | Proportion of population affected by natural disasters five-year average (%) | 0.0361 | 0.018 | No change | Sendai Framework DRR targets are to ‘substantially reduce’ ^[102]^; target set as 30% improvement on baseline value. |
|  | 13.2 Integrate climate change measures into national policies, strategies and planning | 4 | V | 13.2.2 | Total greenhouse gas emissions (excluding LULUCF) (tons CO2-eq) | 540.12m | 324.1 | 83.9 | Target set as 40% improvement on baseline by 2030 from National Sustainable Development Council ^[31]^; and ClimateWorks Australia ^[48]^ and calibrated from baseline data ^[121]^. Target of 85% reduction by 2050. |
|  | 13.2 | 6 | V | 13.2.NEW2 | Greenhouse gas emissions per capita (tons CO2-e /person/year) (excluding LULUCF) | 23.4 | 13.6 | 2.3 | Target set as 40% improvement on baseline from National Sustainable Development Council ^[31]^ and ClimateWorks Australia ^[48]^; calibrated from baseline data ^[121]^. Target of 90% reduction by 2050 to bring close to per capita planetary boundary of 1.6t ^[103]^. |
|  |  |  |  |  |  |  |  |  |  |
| 14 | 14.4 By 2020, effectively regulate harvesting and end overfishing, illegal, unreported and unregulated fishing and destructive fishing practices and implement science-based management plans, in order to restore fish stocks in the shortest time feasible, at least to levels that can produce maximum sustainable yield as determined by their biological characteristics | 6 | V | 14.4.1 | Proportion of fish stocks sustainably exploited (%) | 0.43 | 0.48 | 0.56 | Target set as halt decline and improve by 10% on baseline value by 2030; calibrated from timeseries data (max= 0.85) ^[128, 129]^. In |
|  | 14.5 By 2020, conserve at least 10 per cent of coastal and marine areas, consistent with national and international law and based on the best available scientific information | 6 | V | 14.5.1 | Proportion of territorial waters protected (%) | 0.35 | 0.5 | No change | SDG Target of 10% achieved for Australia ^[101]^. National target set at 50% ; calibrated from timeseries data and BAU projection ^[130, 131]^ |
|  |  |  |  |  |  |  |  |  |  |
| 15 | 15.1 By 2020, ensure the conservation, restoration and sustainable use of terrestrial and inland freshwater ecosystems and their services, in particular forests, wetlands, mountains and drylands, in line with obligations under international agreements | 6 | V | 15.1.1 | Forest cover (as proportion of total land area) (%) | 0.17 | 0.175 | 7% increase | SDG Target 15.1 is ensure conservation, while SDG Target 15.3 is for ‘land degradation neutrality’ or LDN ^[132]^. UN Global Forest Goals include to ‘reverse the loss of forest cover’ ^[133]^. UNCCD guidelines set as no net loss ^[134]^. Target calibrated from timeseries data and BAU projection ^[135]^ and NSDC target for 3.8% increase by 2030 ^[111]^ and ClimateWorks Australia ^[48]^. Target set at 7% increase by 2050. |
|  | 15.1 | 6 | V | 15.1.2 | Proportion of terrestrial area protected (%) | 0.13 | 0.2 | 0.3 | Aichi target of 17% terrestrial areas protected by 2020 ^[136]^. Target set at 20% by 2030 and 30% by 2050; calibrated from timeseries data and BAU projection ^[131]^. |
|  | 15.3 By 2030, combat desertification, restore degraded land and soil, including land affected by desertification, drought and floods, and strive to achieve a land degradation-neutral world | 6 | V | 15.3.1.ALT | Soil Organic Carbon density (dmnl) | 0.00336 | 0.00336 | No change | Target set as maintain or improve based on SDG Target 15.3 which requires ‘no net loss’ ^[101]^ and guidelines from the UN Convention to Combat Desertification on SoC ^[134, 137]^. |
|  | 15.5 Take urgent and significant action to reduce the degradation of natural habitats, halt the loss of biodiversity and, by 2020, protect and prevent the extinction of threatened species | 6 | V | 15.5.1 | Red List Index (score 0-1) | 0.82 | 0.82 | No change | Target 15.5 is to ‘halt loss’. Target set as maintain baseline value; calibrated from timeseries data ^[19, 138, 139]^. |
|  |  |  |  |  |  |  |  |  |  |
| 16 | 16.1 Significantly reduce all forms of violence and related death rates everywhere |  | S | 16.1.1 | Total mortality rate - violence | 0.000022 | 0.00002 | No change | SDG Target 16.1 is to ‘significantly reduce’. Baseline is low for Australia. Target set as maintain or decrease; calibrated from timeseries data ^[130, 140]^. |
|  | 16.5 Substantially reduce corruption and bribery in all their forms |  | S | 16.5.2 | Bribery incidence (score 0-1) | 0.23 | 0.21 | No change | SDG Target 16.5 is to substantially reduce. Baseline is low for Australia. Target set at 10% improvement ^[141]^ |
|  | 16.6 Develop effective, accountable and transparent institutions at all levels |  | S | 16.6.2.ALT1 | Government effectiveness (score -2.5 to +2.5) | 1.6 | 1.9 | No change | Target set at 1.9 on index scale; calibrated from timeseries data and top performing countries (max (2015) = 2.24) ^[8]^ and historic maximum for Australia (2.0). |
|  | 16.6 |  | S | 16.6.2.ALT2 | Political stability (score -2.5 to +2.5) | 0.9 | 1.8 | No change | Target set at 1.8 on index scale; calibrated from timeseries data; data on top performing countries and BAU projection (max (2015) = 1.94) ^[8]^. |
|  | 16.7 Ensure responsive, inclusive, participatory and representative decision-making at all levels |  | S | 16.7.2alt | Normalized average governance index (proxy score 0-1) | 0.8 | 0.82 | No change | Target set at 0.82 on normalized scale (10% improvement); calibrated from BAU projection and World Bank governance data ^[8]^. |
|  |  |  |  |  |  |  |  |  |  |
|  | 17.1 Strengthen domestic resource mobilization, including through international support to developing countries, to improve domestic capacity for tax and other revenue collection |  | E | 17.1.1a | Domestic revenue as share of GDP (%) | 0.344 | 0.344 | No change | SDG Target 17.1 is to strengthen. Target set maintain baseline; calibrated from timeseries data (max = 0.37) ^[18, 19, 142]^. |
|  | 17.1 |  | E | 17.1.2 | Tax burden | 0.65 | <0.65 | No change | Target set at maintain baseline; calibrated off historic data and BAU projection (max = 0.68) ^[18, 19, 109, 142]^. |
|  | 17.1 |  | E | 17.1.2ALT | Government surplus or deficit as proportion GDP (%) | -0.024 | -0.01 | No change | Target set at less than or equal to 1% GDP; calibrated from timeseries data and BAU projection (max = 0.040; min = 0.015) ^[18, 19]^. |
|  | 17.4 Assist developing countries in attaining long-term debt sustainability through coordinated policies aimed at fostering debt financing, debt relief and debt restructuring, as appropriate, and address the external debt of highly indebted poor countries to reduce debt distress |  | E | 17.4.1 | Interest on public debt as share of export (%) | 0.08 | 0.08 | No change | Target set as return to pre-COVID levels; calibrated from timeseries data ^[19, 71]^ and BAU projection. |
|  |  |  |  |  |  |  |  |  |  |

#T= Transformation (T1 to T6)

## D=Dimension: Economic (E): 23; Social (S): 29; Environmental (V): 28

## Supplementary Table 5. Error analysis of baseline (BBS) simulations for a selection of variables

| **Variable** | **R^2^** | **MAPE** |
| --- | --- | --- |
| Total population (persons) | 0.999 | 0.434 |
| Real GDP ($ 2017) | 0.997 | 1.578 |
| Total investment ($) | 0.900 | 21.234 |
| Government Expenditure ($) | 0.999 | 1.429 |
| Government Revenue ($) | 0.999 | 1.497 |
| Total Employment (persons) | 0.996 | 2.255 |
| Total final energy consumption (Ktoe/yr) | 0.954 | 3.655 |
| Domestic material consumption (t) | 0.914 | 3.975 |
| GHGs in CO2-eq (t) | 0.992 | 2.628 |
| Average life expectancy (years) | 0.995 | 1.032 |

## Supplementary Table 6. Sensitivity Analysis (SA): sensitivity variables and input ranges

| **Variable** | **Sensitivity analysis input settings** |
| --- | --- |
| **Average mining gross value added (GVA)** | For mining commodity prices there are several existing Australian and global studies that studies that project changes in total production of key mining commodities, which generally project increased production in commodities through until 2030 ^[9, 26, 143]^. However, these longer-term studies do not explore commodity prices. For the SA, a plausible range was determined based on timeseries data ^[109, 144]^, with the range for SA set at +/-30% of projected average mining gross value added (GVA) by 2050. |
| **Average livestock gross value added (GVA)** | Livestock production projections are calibrated on national economic data ^[145]^ which shows that real production has ranged from 14.5 to 22.6b since 2000. Production values declined from 16.3 to 14.7b in 2020 during the pandemic and following the catastrophic bushfires, a fall of around 10%. The model includes impacts from natural disasters on agricultural capital, however demand for livestock will also be impacted population growth, changing incomes, and shifts in diets. Global projections suggest that demand for meat production will increase by 15% by 2027 ^[146]^. For the purposes of the SA, min/max values were set at +/-30% of projected average livestock gross value added (GVA) by 2050. |
| **Average global temperature** | The SA input range for global average temperatures was based on climate change projections included in the SSPs ^[9]^ as well as CSIRO projections for Australia ^[10]^. Climate results in the SSPs are based on MAGICC 6.8 and values range from 1.552 to 2.527^o^C above pre-industrial levels in 2050, while CSIRO present projections across different RCPs within the range of 0.5 to 3C by 2050. For the purposes of the SA, min/max levels were set at 1-3^o^C by 2050. This range adequately captured the assumptions used in the alternative trajectories (i.e. 1.769 to 1.965^o^C). |
| **Net migration** | The input range for net migration was based on official timeseries data ^[7]^ and population studies available in the literature ^[147-149]^. Net migration is the largest contributor to population growth in Australia. Historic rates of net immigration since 1990 have ranged from approximately 45,000 to 315,000 persons per annum, while modelling studies have used a range from 0 to 1% of total population per annum resulting in a maximum population of 31 million people by 2030. In the model, the trajectories use a net migration target level of 7.8 per thousand people. For the purposes of the SA, min/max levels were set to halve or double this rate by 2050 (3.9 to 15.6). |
| **Required increase in adaptation capital per degree of increase in temp** | Another key assumption in the model relates to the cost of adaptation to climate change impacts. Recent estimates of the cost of climate change impacts on Australia suggest that they will cost Australia $73-94b a year by 2060, up from around $38b in 2020 ^[16]^. However, these costs are uncertain and are estimated as requiring 0.25% GDP of additional capital investment per degree of warming. For the SA, the min/max range was set from 0.05% to 0.5% GDP per degree of warming in required capital investment. This range adequately captures the assumption used in the alternative trajectories (0.25% GDP per degree of warming). |
| **3 x Governance indicators: political stability; government effectiveness; regulatory quality** | Australia’s scores on the five World Bank Governance Indicators are based on global data from the World Bank ^[8]^. Index values for political stability, government effectiveness, and regulatory quality in 2050 are assumed to be higher under the STP trajectory. We test the sensitivity of the results to governance assumptions using min/max values of +/- 30% beyond the ranges used in the trajectories: sdg political stability (0.7 to 2.3), sdg government effectiveness (1.1 to 2.5), sdg regulatory quality (1.3 to 2.6). |
| **Reference interest rate on government domestic and foreign debt** | Interest rates on government debt are estimated in the model based on government finance statistics and interest paid on government debt ^[18, 19]^ however future rates remain highly unertain. Long-term interst rate forecasts for Australia suggest a rate of approximately 3.3% of government bonds maturing in ten years, however historic rates vary from less than 1% to over 6% ^[20]^. We test the sensitivity of results to changes in reference interest rates on both government domestic and foreign debt using min/max values of a halving or doubling in projected long term interest rates (interest rate multiplier of 0.5 to 2). |
| **Average yearly precipitation** | Average year precipitation is estimated in the model based on Australian data and projections global modelling projections ^[11-14]^. Annual average precipitation estimates for 2040-59 range from 549.5mm (SSP1-1.9) to 568.98 (SSP5-8.5). For the purposes of the sensitivity analysis, we use min/max values of +/- 30% in average annual precipitation. |

## References

[**1**] Millennium Institute, *iSDG Model Documentation*. 2022, Millennium Institute: Washington D.C. <https://isdgdoc.millennium-institute.org/en/index.html>

[**2**] Arrow, K.J., *Optimal capital policy, the cost of capital, and myopic decision rules.* Annals of the Institute of Statistical Mathematics, 1964. 16(1): p. 21-30 DOI: <https://doi.org/10.1007/BF02868559>.

[**3**] Lofgren, H., R.L. Harris, and S. Robinson, *A standard computable general equilibrium (CGE) model in GAMS*. 2002, Washington DC, USA: International Food Policy Research Institute.

[**4**] International Monetary Fund, *Government finance statistics manual*. 2014, International Monetary Fund: Washington DC, USA

[**5**] FAO, *Land Cover Classification System: Classification Concepts and User Manual*. 1998, Food and Agriculture Organisation of the United Nations: Rome, Italy

[**6**] International Energy Agency, *Global energy database*. 2019: Paris, France. <https://www.iea.org/statistics/?country=AUSTRALI&year=2016&category=Energy%20supply&indicator=TPESbySource&mode=chart&dataTable=BALANCES>

[**7**] ABS, *3412.0 - Migration, Australia*. 2021, Australian Bureau of Statistics: Canberra, Australia. <https://www.abs.gov.au/AUSSTATS/abs@.nsf/allprimarymainfeatures/D55E16D6BF20AA7CCA2583D00016ED30?opendocument>

[**8**] Kaufman, D. and A. Kraay, *Worldwide Governance Indicators*. 2016, The World Bank: Washington, USA. <http://info.worldbank.org/governance/wgi/index.aspx#home>

[**9**] Riahi, K., et al., *The shared socioeconomic pathways and their energy, land use, and greenhouse gas emissions implications: an overview.* Global Environmental Change, 2017. 42: p. 153-168 DOI: <https://doi.org/10.1016/j.gloenvcha.2016.05.009>.

[**10**] CSIRO, *Climate Futures Exploration Tool*. 2018: Canberra, Australia. <https://www.climatechangeinaustralia.gov.au/en/climate-projections/climate-futures-tool/projections/>

[**11**] BoM, *Annual Climate Statement 2021*. 2021, Australian Bureua of Meteorology: Canberra

[**12**] World Climate Research Programme, *CMIP Phase 6 (CMIP6)*. 2022, World Meteorological Organisation: Geneva

[**13**] World Bank, *Climate Change Knowledge Portal: Mean Projections CMIP6*. 2022, World Bank: Washington

[**14**] FAO, *AQUASTAT*. 2018, Food and Agriculture Organisation of the United Nations: Rome, Italy. <http://www.fao.org/aquastat/en/>

[**15**] Climate Council, *Compound Costs: How climate change is damaging Australia's economy*. 2019, Climate Council of Australia: Canberra. <https://www.climatecouncil.org.au/wp-content/uploads/2019/05/costs-of-climate-change-report-v3.pdf>

[**16**] Deloitte, *Special report: Update to the economic costs of natural disasters in Australia*. 2021, Australian Business Roundtable for Disaster Resilience and Safer Communities: Sydney

[**17**] Climate Council, *Markets are Moving: the Economic Costs of Australia's Climate Inaction*. 2021, Climate Council Australia Ltd: Sydney

[**18**] ABS, *5512.0 - Government Finance Statistics, Australia*. 2021, Australian Bureau of Statistics: Canberra, Australia. <https://www.abs.gov.au/AUSSTATS/abs@.nsf/mf/5512.0>

[**19**] World Bank, *World Development Indicators DataBank*. 2022: Washington, USA. <https://data.worldbank.org/indicator/SP.DYN.CONU.ZS?view=chart>

[**20**] OECD, *Long-term interest rates forecast*. 2022, Organisation for Economic Cooperation and Development: Paris

[**21**] Rogelj, J., et al., *Mitigation pathways compatible with 1.5 C in the context of sustainable development*, in *Global warming of 1.5 C*. 2018, Intergovernmental Panel on Climate Change: Geneva. p. 93-174.

[**22**] Gidden, M.J., et al., *Global emissions pathways under different socioeconomic scenarios for use in CMIP6: a dataset of harmonized emissions trajectories through the end of the century.* Geoscientific model development, 2019. 12(4): p. 1443-1475 DOI: <https://doi.org/10.5194/gmd-12-1443-2019>.

[**23**] IEA, *Net Zero by 2050: A Roadmap for the Global Energy Sector*. 2021, International Energy Agency: Paris

[**24**] IPCC, *Climate Change 2022 Mitigation of Climate Change*, in *Working Group III contribution to the Sixth Assessment Report of the IPCC*. 2022, Intergovernmental Panel on Climate Change: Geneva

[**25**] Climateworks Centre and Climate-KIC Australia, *Pathways to industrial decarbonisation: positioning Australian industry to prosper in a net zero global economy*. 2023, Australian Industy Energy Transition Initiative: Melbourne

[**26**] Resources 2030 Taskforce, *Australian resources - providing prosperity for future generations*. 2018, Commonwealth of Australia: Canberra, Australia

[**27**] ABS, *Australian Industry*, A.B.o. Statistics, Editor. 2022: Canberra

[**28**] National Resilience Taskforce, *Profiling Australia's Vulnerability*, in *The interconnected causes and cascading effects of systemic disaster risk*. 2018, Commonwealth of Australia: Canberra

[**29**] Department of Health, *National Preventitive Health Strategy 2021-2030*, in *Valuing health before illness: Living well for longer*. 2021, Australian Government: Canberra

[**30**] Allen, C., et al., *Transforming Austrlaia SDG Progress Report - 2020 Update*, in *Australia's Progress and Potential Impacts from COVID-19*. 2020, Monash University: Melbourne

[**31**] Allen, C., et al., *Assessing national progress and priorities for the Sustainable Development Goals (SDGs): experience from Australia.* Sustainability Science, 2019. 15: p. 521-538 DOI: <https://doi.org/10.1007/s11625-019-00711-x>.

[**32**] NSW Government, *A wellbeing budget for NSW*, in *Foundation PAper*. 2022, NSW Department of Planning and Environment Sydney

[**33**] PwC, *Where next for Australia's tax system?*, in *How our tax system can help reboot prosperity for Australia*. 2020, Price Waterhouse Coopers: Sydney

[**34**] ACOSS and UNSW Sydney, *Inequality in Australia 2020 Supplement: The impact of COVID-19 on income inequality*. 2020, ACCOSS: Sydney

[**35**] BZE, *Export Powerhouse: Australia's $333 billion opportunity*. 2021, Beyond Zero Emissions: Melbourne

[**36**] ACOSS and UNSW Sydney, *Poverty In Australia 2020*. 2020, Australian Council of Social Services: Sydney

[**37**] BZE, *The Million Jobs Plan*. 2020, Beyond Zero Emissions: Melbourne. <https://bze.org.au/wp-content/uploads/2020/11/BZE-The-Million-Jobs-Plan-Full-Report-2020.pdf>

[**38**] Jones, A., et al., *Integrating wellbeing into the business of government: The feasibility of innovative legal and policy measures to achieve sustainable development in Australia*. 2021, Victorian Health Promotion Foundation and the George Institute for Global Health: Melbourne

[**39**] ASFI, *Australian Sustainable Finance Roadmap: a plan for aligning Australia's financial system with a sustainable, resilient and prosperous future for all Australians*. 2020, Australian Sustainable Finance Inititative: Melbourne

[**40**] ACSI, *ESG Reporting Trends*, in *A detailed assessment of ESG reporting in ASX200 companies*. 2022, Australian Council of Superannuation Investors: Melbourne

[**41**] AEGN, *Sustainable Food Systems*, in *Philanthropy Briefing*. 2019, Australian Environmental Grantmakers Network: Melbourne

[**42**] Food Security and Food Systems Working Group, *Towards a Healthy Regenerative and Equitable Food System in Victoria: Consensus Statement*. 2022, VicHealth: Melbourne

[**43**] FOLU, *Accelerating the 10 Critical Transitions: Positive Tipping Points for Food and Land Use Systems Transformation*. 2021, Food and Land Use Coalition and Global Systems Institute: UK. <https://www.foodandlandusecoalition.org/wp-content/uploads/2021/07/Positive-Tipping-Points-for-Food-and-Land-Use-Systems-Transformation.pdf>

[**44**] Frison, E.A., *From uniformity to diversity: a paradigm shift from industrial agriculture to diversified agroecological systems.* 2016.

[**45**] Iles, A., *Can Australia transition to an agroecological future?* Agroecology and Sustainable Food Systems, 2021. 45(1): p. 3-41.

[**46**] Lawrence, G., C. Richards, and K. Lyons, *Food security in Australia in an era of neoliberalism, productivism and climate change.* Journal of Rural Studies, 2013. 29: p. 30-39 DOI: <https://doi.org/10.1016/j.jrurstud.2011.12.005>.

[**47**] Burch, D., G. Lawrence, and L. Hattersley, *Watchdogs and ombudsmen: monitoring the abuse of supermarket power.* Agriculture and Human Values, 2013. 30(2): p. 259-270 DOI: <https://doi.org/10.1007/s10460-012-9412-8>.

[**48**] ClimateWorks Australia, *Decarbonisation Futures: Solutions, actions and benchmarks for a net zero emissions Australia*. 2020, ClimateWorks: Melbourne

[**49**] Herrero, M., et al., *Articulating the effect of food systems innovation on the Sustainable Development Goals.* The Lancet Planetary Health, 2021. 5(1): p. e50-e62 DOI: <https://doi.org/10.1016/S2542-5196(20)30277-1>.

[**50**] Herrero, M., et al., *Innovation can accelerate the transition towards a sustainable food system.* Nature Food, 2020. 1(5): p. 266-272 DOI: <https://doi.org/10.1038/s43016-020-0074-1>.

[**51**] ClimateWorks Centre, *Living within limits: Adapting the planetary boundaries to understand Australia's contribution to planetary health: Technical report*. 2022, ClimateWorks Centre: Melbourne

[**52**] Anderson, C.R., et al., *From transition to domains of transformation: Getting to sustainable and just food systems through agroecology.* Sustainability, 2019. 11(19): p. 5272 DOI: <https://doi.org/10.3390/su11195272>.

[**53**] Hadjikakou, M. and T. Wiedmann, *Shortcomings of a growth-driven food system*, in *Handbook on Growth and Sustainability*. 2017, Edward Elgar Publishing. p. 256-276.

[**54**] Hudson, M., *Enacted inertia: Australian fossil fuel incumbents’ strategies to undermine challengers*, in *The Palgrave Handbook of Managing Fossil Fuels and Energy Transitions*. 2020, Springer. p. 195-222.

[**55**] AER, *State of the Energy Market 2021*. 2021, Australian Energy Regulator: Canberra

[**56**] Future Earth, *Sustainable Cities and Regions*, in *10 year strategy to enable urban systems transformation*. 2019, Australian Academy of Sciences: Canberra

[**57**] Newton, P., et al., *Decarbonising the built environment: charting the transition*. 2019: Springer.

[**58**] Candy, S., et al., *Results from Visions and Pathways 2040: Scenarios and Pathways to Low Carbon Living*. 2017, CRC for Low Carbon Living: Melbourne

[**59**] Webb, R., et al., *Sustainable urban systems: Co-design and framing for transformation.* Ambio, 2018. 47(1): p. 57-77 DOI: <https://doi.org/10.1007/s13280-017-0934-6>.

[**60**] Sharpe, S. and T.M. Lenton, *Upward-scaling tipping cascades to meet climate goals: Plausible grounds for hope.* Climate Policy, 2021. 21(4): p. 421-433 DOI: <https://doi.org/10.1080/14693062.2020.1870097>.

[**61**] Australian Government, *Australia State of the Environment 2021*. 2021, Commonwealth of Australia: Canberra

[**62**] Commonwealth of Australia, *Australia's Strategy for Nature 2019-2030*. 2019, Commonwealth of Australia: Canberra. <https://www.australiasnaturehub.gov.au/sites/default/files/2020-11/australias-strategy-for-nature.pdf>

[**63**] Coffey, B., et al., *Assessing biodiversity policy designs in Australia, France and Sweden. Comparative lessons for transformative governance of biodiversity?* Journal of Environmental Policy & Planning, 2022: p. 1-14 DOI: <https://doi.org/10.1080/1523908X.2022.2117145>.

[**64**] ClimateWorks, *Natural Capital Roadmap*. 2019, ClimateWorks: Melbourne

[**65**] Australian Treasury, *Economic Response to COVID-19*. 2022, Australian Government: Canberra. <https://treasury.gov.au/coronavirus>

[**66**] Australian Treasury, *Budget 2020-21: Budget Strategy and Outlook*. 2020, Australian Government: Canberra

[**67**] OECD, *OECD National Accounts Statistics: General government spending*. 2019: Paris, France. <https://data.oecd.org/gga/general-government-spending.htm>

[**68**] OECD, *OECD Social and Welfare Statistics*, O.f.E.C.a. Development, Editor. 2018: Paris, France. <https://www.oecd-ilibrary.org/social-issues-migration-health/data/social-expenditure/aggregated-data_data-00166-en>

[**69**] ABS, *6523.0 - Household Income and Wealth, Australia*. 2018, Australian Bureau of Statistics: Canberra, Australia. <https://www.abs.gov.au/AUSSTATS/abs@.nsf/allprimarymainfeatures/B3AB9C8CD32F7CA6CA2584340018A13B?opendocument>

[**70**] Melbourne Institute, *The Household, Income and Labour Dynamics in Australia (HILDA) Survey: Selected Findings from Waves 1 to 16*. 2018, Melbourne Institute: Melbourne, Australia. <https://melbourneinstitute.unimelb.edu.au/__data/assets/pdf_file/0009/2874177/HILDA-report_Low-Res_10.10.18.pdf>

[**71**] IMF, *Government Finance Statistics*, I.M. Fund, Editor. 2019: Washington, USA. <http://data.imf.org/?sk=a0867067-d23c-4ebc-ad23-d3b015045405>

[**72**] BITRE, *Australian Infrastructure Statistics Yearbook 2016*. 2016, Australian Government Bureau of Infrastructure, Transport and Regional Economies: Canberra, Australia. <https://www.bitre.gov.au/publications/2016/yearbook_2016.aspx>

[**73**] Nahum, D., *Powering Onwards: Australia's Opportunity to Reinvigorate Manufacturing through Renewable Energy*. 2020, The Centre for Future Work: Canberra

[**74**] West, J. and M. Lieber, *Global Material Flows Database*. 2018: Paris, France. <https://www.resourcepanel.org/global-material-flows-database>

[**75**] Hickey, M., et al., *Maximising returns from water in the Australian vegetable industry: national report*. 2006, NSW Department of Primary Industries: Sydney

[**76**] Government of Victoria, *Farm Water Fact Sheet*. 2015, Government of Victoria: Melbourne

[**77**] FAO, *FAOSTAT database*. 2017: Rome, Italy. <http://www.fao.org/faostat/en/#data/RL>

[**78**] ABS, *6202.0 - Labour Force, Australia*. 2017, Australian Bureau of Statistics: Canberra, Australia. <https://www.abs.gov.au/AUSSTATS/abs@.nsf/allprimarymainfeatures/DAFD6C38CD421642CA25819A0013343A?opendocument>

[**79**] Graham, P., et al., *Updated projections of electricity generation technology costs*. 2018, CSIRO: Newcastle

[**80**] Hayward, J. and P. Graham, *Electricity generation technology cost projections, 2017-2050*. 2017, CSIRO: Canberra, Australia

[**81**] The Climate Institute, *Australia's National Strategy for Energy Efficiency*. 2008, The Climate Institute: Canberra

[**82**] Commonwealth of Australia, *Improving the efficiency of new light vehicles*. 2016, Australian Government: Canberra,

[**83**] ASBEC, *Low Carbon High Performance: How buildings can make a major contribution to Australia’s emissions and productivity goals*. 2016, Australian Sustainable Built Environment Council: Melbourne

[**84**] Allen, C., et al., *Modelling ambitious climate mitigation pathways for Australia's built environment.* Sustainable Cities and Society, 2021. 77(102554) DOI: <https://doi.org/10.1016/j.scs.2021.103554>.

[**85**] Skullestad, J.L., R.A. Bohne, and J. Lohne, *High-rise timber buildings as a climate change mitigation measure–A comparative LCA of structural system alternatives.* Energy Procedia, 2016. 96: p. 112-123 DOI: <https://doi.org/10.1016/j.egypro.2016.09.112>.

[**86**] Wood Solutions, *Environmental Product Declarations*. 2017, Forest and Wood Products Australia Ltd: Melbourne. <https://www.woodsolutions.com.au/articles/environmental-product-declarations>

[**87**] Australian Government, *Australian Waste Policy Action Plan*. 2019, Commonwealth of Australia: Canberra

[**88**] Energeia, *Australian Electric Vehicle Market Study: Report prepared for the Australian Renewable Energy Agency and the Clean Energy Finance Corporation*. 2018, Australian Government: Canberra, Australia

[**89**] Energeia, *Electric Vehicles Insights: Prepared for the Australian Energy Market Operator's 2017 Electricity Forecast Insights*. 2017, Energeia: Canberra

[**90**] Broadbent, G., et al., *The role of electric vehicles in decarbonising Australia’s road transport sector: modelling ambitious scenarios.* Energy Policy, 2022. 168: p. 113144 DOI: <https://doi.org/10.1016/j.enpol.2022.113144>.

[**91**] Broadbent, G.H., et al., *Accelerating electric vehicle uptake: Modelling public policy options on prices and infrastructure.* Transportation Research Part A: Policy and Practice, 2022. 162: p. 155-174 DOI: <https://doi.org/10.1016/j.tra.2022.05.012>.

[**92**] ABS, *Motor Vehicle Census, Australia, 2020*. 2020, Australian Bureau of Statistics: Canberra

[**93**] ABS, *Survey of Motor Vehicle Use, Australia*, in *9208.0*. 2020, Australian Bureau of Statistics: Canberra

[**94**] Summers, D.M., et al., *The costs of reforestation: a spatial model of the costs of establishing environmental and carbon plantings.* Land Use Policy, 2015. 44: p. 110-121 DOI: <https://doi.org/10.1016/j.landusepol.2014.12.002>.

[**95**] Australian Government. *National Landcare Program: 20 Million Trees Program*. 2018 10 June 2018]; Available from: <http://www.nrm.gov.au/national/20-million-trees>.

[**96**] Balmford, A., et al., *The worldwide costs of marine protected areas.* Proceedings of the National Academy of Sciences, 2004. 101(26): p. 9694-9697 DOI: <https://doi.org/10.1073/pnas.0403239101>.

[**97**] Ban, N.C., et al., *Promise and problems for estimating management costs of marine protected areas.* Conservation Letters, 2011. 4(3): p. 241-252 DOI: <https://doi.org/10.1111/j.1755-263X.2011.00171.x>.

[**98**] Adams, V.M., D.B. Segan, and R.L. Pressey, *How much does it cost to expand a protected area system? Some critical determining factors and ranges of costs for Queensland.* PloS one, 2011. 6(9): p. e25447.

[**99**] James, A., J. Green, and J. Paine, *A Global Review of Protected Area Budgets and Staff*. 1999, World Conservation Monitoring Centre: Cambridge, U.K. <https://www.infra.cbd.int/financial/expenditure/g-spendingglobal-wcmc.pdf>

[**100**] ABS, *4183.0 - Cultural Funding by Government, Australia*. 2014, Australian Bureau of Statistics: Canberra, Australia. <https://www.abs.gov.au/AUSSTATS/abs@.nsf/Latestproducts/4183.0Main%20Features32012-13?opendocument&tabname=Summary&prodno=4183.0&issue=2012-13&num=&view>=

[**101**] UNGA, *Transforming our world: the 2030 Agenda for Sustainable Development, outcome document of the United Nations summit for the adoption of the post-2015 agenda*, in *RES/A/70/L.1.* 2015, United Nations General Assembly: New York

[**102**] United Nations, *Sendai Framework for Disaster Risk Reduction 2015-2030*. 2015, United Nations Office for Disaster Risk Reduction: New York. <https://www.unisdr.org/we/inform/publications/43291>

[**103**] O’Neill, D.W., et al., *A good life for all within planetary boundaries.* Nature Sustainability, 2018. 1(2): p. 88 DOI: <https://doi.org/10.1038/s41893-018-0021-4>.

[**104**] Australian Institute of Health & Welfare, *Teenage mothers in Australia*. 2018, Australian Government: Canberra, Australia. <https://www.aihw.gov.au/reports/mothers-babies/teenage-mothers-in-australia-2015/contents/table-of-contents>

[**105**] OECD, *Education Database - Education at a Glance*. 2017: Paris, France. <https://data.oecd.org/students/enrolment-rate-in-secondary-and-tertiary-education.htm>

[**106**] Barro, R. and J. Lee, *A New Data Set of Educational Attainment in the World, 1950-2010.* Journal of Development Economics, 2013. 104: p. 184-198 DOI: <https://doi.org/10.1016/j.jdeveco.2012.10.001>.

[**107**] ABS, *4610.0 - Water Account, Australia*. 2017, Australian Bureau of Statistics: Canberra, Australia. <https://www.abs.gov.au/AUSSTATS/abs@.nsf/DetailsPage/4610.02015-16>

[**108**] OECD, *OECD Environmental Outlook to 2050: The consequences of inaction*. 2012, Organisation for Economic Cooperation and Development: Paris

[**109**] ABS, *5204.0 - Australian System of National Accounts*. 2017, Australian Bureau of Statistics: Canberra, Australia. <https://www.abs.gov.au/AUSSTATS/abs@.nsf/allprimarymainfeatures/D287F7C09246B5BDCA258331000C3A24?opendocument>

[**110**] ABS, *3101.0 - Australian Demographic Statistics*. 2017, Australian Bureau of Statistics: Canberra, Australia. <https://www.abs.gov.au/AUSSTATS/abs@.nsf/mf/3101.0>

[**111**] MSDI, *Transforming Australia 2020 Update*. 2020, Monash Sustainable Development Institute: Melbourne

[**112**] DEE, *Australian Energy Statistics*. 2019, Australian Government: Canberra, Australia. <https://www.energy.gov.au/government-priorities/energy-data/australian-energy-statistics>

[**113**] ABS, *4604.0 - Energy Account, Australia*. 2018, Australian Bureau of Statistics: Canberra, Australia. <https://www.abs.gov.au/ausstats/abs@.nsf/mf/4604.0>

[**114**] Council of Australian Governments, *National Energy Productivity Plan 2015-2030*. 2015: Canberra, Australia. <http://www.coagenergycouncil.gov.au/publications/national-energy-productivity-plan-2015-2030>

[**115**] ABS, *5206.0 - Australian National Accounts: National Income, Expenditure and Product*. 2020, Australian Bureau of Statistics: Canberra, Australia. <https://www.abs.gov.au/ausstats/abs@.nsf/mf/5206.0>

[**116**] International Resource Panel, *Resource Efficiency: Potential and Economic Implications.* . 2017, UN Environment: Paris

[**117**] BITRE, *Road Construction Cost and Infrastructure Procurement Benhmarking: 2017 Update*. 2017, Australian Government Bureau of Infrastructure, Transport and Regional Economies: Canberra, Australia. <https://www.bitre.gov.au/publications/2018/rr_148.aspx>

[**118**] Gargett, D. *Costs of the Austalian Road System*. in *Australasian Transport Research Forum 2017 Proceedings*. 2017. Auckland, New Zealand.

[**119**] BITRE, *Trainline 5*. 2017, Australian Government Bureau of Infrastructure, Transport and Regional Economies: Canberra, Australia. <https://www.bitre.gov.au/publications/2017/files/train_005.pdf>

[**120**] ABS, *8155.0 - Australian Industry*. 2018, Australian Bureau of Statistics: Canberra, Australia. <https://www.abs.gov.au/AUSSTATS/abs@.nsf/allprimarymainfeatures/ECC05829CF35B0CCCA25840A001811F6?opendocument>

[**121**] Australian Department of the Environment and Energy, *Australia's emissions projections 2017*. 2017, Australian Government: Canberra, Australia. <http://www.environment.gov.au/system/files/resources/eb62f30f-3e0f-4bfa-bb7a-c87818160fcf/files/australia-emissions-projections-2017.pdf>

[**122**] OECD, *OECD.stat Green Growth Indicators*. 2017, Organisation for Economic Cooperation and Development. <https://stats.oecd.org/Index.aspx?DataSetCode=GREEN_GROWTH>

[**123**] Schmidt-Traub, G., et al., *National baselines for the Sustainable Development Goals assessed in the SDG Index and Dashboards.* Nature Geoscience, 2017. 10(8) DOI: <https://doi.org/10.1038/ngeo2985>.

[**124**] Sachs, J., et al., *SDG Index and Dashboards Report 2017.* Bertelsmann Stiftung and Sustainable Development Solutions Network (SDSN): New York, NY, USA, 2017.

[**125**] NTC, *VFACTS National EV PHEV Hybrid Sales 2011-20.* 2020

[**126**] WHO, *WHO Air quality guidelines for particulate matter, ozone, nitrogen dioxide and sulfur dioxide*. 2005, World Health Organisation: Switzerland. <https://apps.who.int/iris/bitstream/handle/10665/69477/WHO_SDE_PHE_OEH_06.02_eng.pdf;sequence=1>

[**127**] Sachs, J., et al., *The Decade of Action for the Sustainable Development Goals: Sustainable Development Report 2021*. 2021, Cambridge University Press: Cambridge. <https://www.sustainabledevelopment.report/>

[**128**] Kleisner, K., et al., *Australia: Reconstructing estimates of total fisheries removal, 1950-2010*. 2015, University of British Columbia: British Columbia. <http://www.seaaroundus.org/data/#/eez/36?chart=catch-chart&dimension=taxon&measure=tonnage&limit=10>

[**129**] Pauly, D. and D. Zeller, *Seas Around Us Concepts, Design and Data*. 2015, University of British Columbia: British Columbia. <http://www.seaaroundus.org>

[**130**] United Nations, *United Nations Global SDG Database*. 2018, United Nations: New York. <https://unstats.un.org/sdgs/indicators/database/>

[**131**] IUCN and UNEP-WCMC, *World Database on Protected Areas*. 2017, International Union for the Conservation of Nature and United Nations Environment Programme World Conservation Monitoring Centre. <https://www.iucn.org/theme/protected-areas/our-work/world-database-protected-areas>

[**132**] United Nations Development Group, *Mainstreaming the 2030 Agenda for Sustainable Development: Interim Reference Guide to UN Country Teams*. 2015

[**133**] United Nations, *United Nations Strategic Plan for Forests 2017-2030*. 2017, United Nations: New York. <https://documents-dds-ny.un.org/doc/UNDOC/GEN/N17/184/62/PDF/N1718462.pdf?OpenElement>

[**134**] Orr, B., et al. *Scientific conceptual framework for land degradation neutrality*. in *Bonn, Germany: United Nations Convention to Combat Desertification (UNCCD)*. 2017.

[**135**] ABARES, *Australia's State of the Forests Report 2013*. 2013, Australian Bureau of Agriculture and Resource Economics and Sciences, Commonwealth of Australia: Canberra, Australia. <http://www.agriculture.gov.au/abares/forestsaustralia/Pages/SOFR/sofr-2013.aspx>

[**136**] United Nations Convention on Biological Diversity, *Strategic Plan for Biodiversity 2011-2020*. 2010, United Nations: Montreal, Canada. <https://www.cbd.int/decision/cop/?id=12268>

[**137**] Cowie, A.L., et al., *Land in balance: The scientific conceptual framework for Land Degradation Neutrality.* Environmental Science & Policy, 2018. 79: p. 25-35 DOI: <https://doi.org/10.1016/j.envsci.2017.10.011>.

[**138**] Global Environment Facility, *Technical Paper on the GEF Resource Allocation Framework*. 2005, Global Environment Facility: Washington DC, USA. <https://www.thegef.org/sites/default/files/council-meeting-documents/C.26.2.Rev_.1_Technical_Note_on_RAF_5.pdf>

[**139**] Pandey, K.D., et al., *Biodiversity Conservation Indicators: New Tools for Priority-Setting at the Global Environment Facility*, in *World Bank Development Research Group Working Paper*. 2006, World Bank: Washington DC

[**140**] WHO, *Global Health Estimates: Disease burden by Cause, Age, Sex, Country and Region*. 2018, World Health Organisation: Switzerland. <https://www.who.int/healthinfo/global_burden_disease/estimates/en/index1.html>

[**141**] Transparency International, *Corruption Perception Index*. 2016, Transparency International: Berlin. <https://www.transparency.org/research/cpi/overview>

[**142**] OECD, *OECD.stat - Public Sector, Taxation and Market Regulation*. 2018, Organisation for Economic Cooperation and Development: Paris, France. <https://stats.oecd.org/Index.aspx?DataSetCode=REV>

[**143**] Syed, A., et al., *Australian energy projections to 2029-30, ABARE research report 10.02, prepared for the Department of Resources, Energy and Tourism*. 2010, ABARES: Canberra

[**144**] ABS, *8415.0 - Mining Operations, Australia*. 2017, Australian Bureau of Statistics: Canberra, Australia. <https://www.abs.gov.au/ausstats/abs@.nsf/0/D96FCC4AEEA50923CA2568A90013940B?Opendocument>

[**145**] ABS, *Australian System of National Accounts*, in *ABS Catalogue Number 5204.0*. 2021, Australian Bureau of Statistics: Canberra. <https://www.abs.gov.au/statistics/economy/national-accounts/australian-system-national-accounts>

[**146**] OECD and FAO, *OECD-FAO Agricultural Outlook 2018-2027*. 2017, Organisation for Economic Cooperation and Development and the UN Food and Agricultural Organisation.: Rome, Italy

[**147**] Sobels, J., et al., *Research into the Long-Term Physical Implications of Net Overseas Migration*. 2010, ational Institute of Labour Studies, Flinders University School of the Environment, and CSIRO Sustainable Ecosystems: Adelaide, Australia

[**148**] McDonald, P.F. and J. Temple, *Immigration, labour supply and per capita Gross Domestic Product: Australia 2010-2050*. 2010, Canberra, Australia: Australian Government Department of Immigration and Citizenship.

[**149**] Australian Productivity Commission, *Migrant Intake to Australia - Productivity Commission Inquiry Report*. 2016, Australian Government: Canberra, Australia
