# Supplementary material for: Modelling six sustainable development transformations in Australia and their accelerators, impediments, enablers, and interlinkages
Source: Nat Commun. 2024 Jan 18;15:594. doi: 10.1038/s41467-023-44655-4 (PMC10796343; doi:10.1038/s41467-023-44655-4)
Supplement: Supplementary file 3 — Description of Additional Supplementary Files [file 41467_2023_44655_MOESM3_ESM.pdf]

Description of Additional Supplementary Files

File Name: Supplementary Data 1
Description: This file contains additional supplementary datasets to support the manuscript: 1. Model_Data includes the input dataset used to calibrate the iSDG-Australia 2.0 system dynamics model; and 2. Model_Projections_2050 provides the model projections from 2020 to 2050 for the two modelled pathways across a range of key indicators (BBS=Build Back the Same Pathway and represents the baseline scenario; STP=Six Transformations Pathway).
